# On the development of an information system for monitoring user opinion and its role for the public

**DOI:** 10.1186/s40537-022-00660-w

**Published:** 2022-11-21

**Authors:** Vladislav Karyukin, Galimkair Mutanov, Zhanl Mamykova, Gulnar Nassimova, Saule Torekul, Zhanerke Sundetova, Matteo Negri

**Affiliations:** 1 Al-Farabi Kazakh National University, Almaty, 050040 Kazakhstan; 2 Fondazione Bruno Kessler, 38123 Trento, Italy

**Keywords:** Social media, Social networks, Social mood, Sentiment analysis, Analytics platforms, Sentiment dictionary, Machine learning, OMSystem, Coronavirus disease, Vaccination

## Abstract

Social media services and analytics platforms are rapidly growing. A large number of various events happen mostly every day, and the role of social media monitoring tools is also increasing. Social networks are widely used for managing and promoting brands and different services. Thus, most popular social analytics platforms aim for business purposes while monitoring various social, economic, and political problems remains underrepresented and not covered by thorough research. Moreover, most of them focus on resource-rich languages such as the English language, whereas texts and comments in other low-resource languages, such as the Russian and Kazakh languages in social media, are not represented well enough. So, this work is devoted to developing and applying the information system called the OMSystem for analyzing users’ opinions on news portals, blogs, and social networks in Kazakhstan. The system uses sentiment dictionaries of the Russian and Kazakh languages and machine learning algorithms to determine the sentiment of social media texts. The whole structure and functionalities of the system are also presented. The experimental part is devoted to building machine learning models for sentiment analysis on the Russian and Kazakh datasets. Then the performance of the models is evaluated with accuracy, precision, recall, and F1-score metrics. The models with the highest scores are selected for implementation in the OMSystem. Then the OMSystem’s social analytics module is used to thoroughly analyze the healthcare, political and social aspects of the most relevant topics connected with the vaccination against the coronavirus disease. The analysis allowed us to discover the public social mood in the cities of Almaty and Nur-Sultan and other large regional cities of Kazakhstan. The system’s study included two extensive periods: 10-01-2021 to 30-05-2021 and 01-07-2021 to 12-08-2021. In the obtained results, people’s moods and attitudes to the Government’s policies and actions were studied by such social network indicators as the level of topic discussion activity in society, the level of interest in the topic in society, and the mood level of society. These indicators calculated by the OMSystem allowed careful identification of alarming factors of the public (negative attitude to the government regulations, vaccination policies, trust in vaccination, etc.) and assessment of the social mood.

## Introduction

The rapid development of the Internet, social networks, online services, and other web resources have initiated a great interest in the use of information from social networks and the great online activity of users. Research on social media platforms has shown a significant increase in the number of users over the last decade [1]. Older social media platforms like Facebook, YouTube, Reddit, Twitter, etc., save their popularity and are replenished by an even greater number of users. Meanwhile, new platforms, such as Instagram, Tumblr, TikTok, Pinterest, and others, are strengthening their positions in the media space every year [2]. These platforms have been developing not only in the entertainment direction but also in other spheres of life as new events occur almost daily, and their relevance is constantly changing.

In many cases, social networks are used to solve a wide range of business tasks: managing and promoting brands [3], advertising goods and services, creating distribution channels for goods, etc. In addition to business tasks [4], there is a great need for monitoring social networks [5] and content analysis in other areas. Critical topics in politics [6], economics [7], healthcare, medicine, culture, and other areas are gaining great popularity in the media space [8]. It is possible to get the results of public opinion on various social and political topics from discussion places on social networks. In this regard, the technologies of “monitoring social networks” (social listening) and content analysis are gaining great popularity. The number of analytics platforms has significantly increased in the last few years. The lists of the most popular platforms can be easily found online with descriptions of their features and characteristics. Sproutsocial, Hubspot, Buzzsumo, Hootsuite, Brandmention, IQBuzz, and Snaplytics are good examples of such analytics applications. The description of features and characteristics of these platforms are thoroughly described in “Analytics platforms” section of this research. Despite a large number of such platforms, they remind each other in a way that they immensely focus on business purposes leaving significant social, economic, and political problems uncovered. Moreover, all of them are not open access and require a regular paid subscription for their full service. The majority of published papers in reputable journals are devoted to sentiment analysis (SA) of user comments from the Twitter social network [9–11]. The research topic of many papers also covers the presidential elections in the USA [9, 10] and other countries [11, 12]. At the same time, the works studying and describing complex social analytics platforms, such as [13], are not fully presented.

Moreover, most of them focus on resource-rich languages such as English, German, French, Italian, Spanish, and Portuguese languages, whereas texts and comments in other low-resource languages such as Russian and Kazakh languages are underrepresented. The web crawlers of the platforms are also not configured to extract texts from the social media space of Kazakhstan. This problem is significant for Kazakhstan, where social media content is mostly written in Russian and Kazakh languages. In addition, it is essential to receive information about current topics in the country from the most popular news portals and discussion platforms on social networks. Even though the news portals tend to publish their content in both languages, it has been noticed during the manual analysis of parsed texts that user comments in Russian prevail over comments in Kazakh, which makes obtaining data even more valuable for understanding the sentiments of the Kazakh speaking population of the country.

Thereby, a new opinion monitoring information system, the OMSystem, which pays much attention to the political, economic, healthcare, education, culture, ecology, and civil society topics, has been developed. This multifunctional platform monitors the media space of Kazakhstan and supports the Kazakh and Russian languages, which allows analyzing the media space efficiently. The OMSystem supports Kazakhstan’s leading news portals and important popular social networks like Facebook, VKontakte, Instagram, Twitter, and YouTube. The core part of the system is the evaluation of the public’s mood and “social well-being” with the use of the SA tool and the social mood indicators such as the level of topic discussion activity in society, the level of interest in the topic in society, and the level of social mood. The SA tool determines the sentiment [14] of the public mood, the range of interests, and information dissemination. It also identifies current problematic issues in society and tracks the dynamics of user involvement in a certain topic. This tool uses the SA methods generally presented by three main approaches: lexicon-based, machine learning-based, and deep learning-based.

This paper describes the architecture of the OMSystem, main modules, and functionalities of this platform, focusing on the SA tools and the module for defining the social mood of society. The use of sentiment dictionaries as a lexicon-based approach and machine learning (ML) algorithms in the OMSystem are also carefully explained. The first part of the experimental section presents the steps to train ML models and select the most efficient ones for use in the OMSystem. The second part demonstrates the definition of the public opinion on the topic of vaccination against coronavirus infection by the evaluation with the following social mood estimating measures: the level of topic discussion activity in society, the level of interest in the topic in society, and the level of social mood. Many scientific articles review the topics related to the Covid-19 pandemic, and research in this field is especially demanded today. Nevertheless, most of the papers were devoted to analyzing labeled sentiment texts, posts, and tweets from social media platforms to evaluate the ML metrics of the trained models. Still, they did not summarize texts together to use other social measures to provide the general people’s attitudes towards the different aspects of this critical topic [15, 16]. Thara and Poornachandran [17] focuses on building SA models with ML algorithms and estimating social mood with the abovementioned measures. The developed ML models have been evaluated by accuracy, precision, recall, and F1-score measures to find the most effective algorithms that need to be used in the OMSystem. The social mood part has also provided exciting findings about the public’s attitude to the vaccination campaign, vaccination policies, and the Government’s activities and methods of combating the pandemic. The reasons for people’s negative moods on this topic have also been extensively analyzed.

The rest of the paper is organized in the following way: “Related works” section provides an overview of the related works to this paper. “Analytics platforms” section describes the features of popular social analytics platforms for brand monitoring, highlighting the essential missing tools implemented in the OMSystem. “The OMSystem information system design methodology” and “The linguistic module” sections describe the structure, functionalities, and module for SA and social mood evaluation. “Machine learning methods” section describes and discusses the experiments on the development of ML algorithms and the public’s attitude towards the vaccination against coronavirus infection. Finally, in “Data collection and data processing” section, we summarize all the previously described sections, analyze the obtained results, and outline directions for future research.

## Related works

In recent years, the active development of web technologies has made it possible to analyze users’ moods on various topics. At the same time, marketing campaigns interested in learning users’ opinions and developing many strategies for increasing the flow of customers and profits play a significant role in data analytics. The manual search and filtering of users’ views on websites remain challenging because of their vast number. Therefore, special tools have been developed to automatically track, summarize, and visualize information from social content to solve this problem. In [18], SA of the popular smartphone brand was presented. Data was collected from Twitter using a web crawler that searches through particular hashtags. Benedetto and Tedeschi [19] demonstrates an open framework for monitoring, analyzing, and receiving media content. This framework allows you to collect, index, and retrieve data using the Representational state transfer application programming interface (REST API) from the following sources: Twitter, Facebook, YouTube, Google+, and Flickr. Schinas et al. [20] presents an analysis of the statements of many political leaders, diplomats, journalists, and other media figures on the Twitter platform, the most active social network covering these issues. Radicioni et al. [21] shows an architecture that combines SA and community discovery to understand trends, approaches, business, and policy views on topics such as shopping, politics, Covid-19, and electric vehicles. At the same time, many works are devoted to describing analytics platforms, social networks, and text processing for SA. Bhatnagar and Choubey [22] describes the steps of preprocessing, vectorization, and classification of the textual data using ML algorithms. Nandwani and Verma [23] pays great attention to studying the critical approaches of the most efficient ML algorithms for SA. That work showed that the Support vector machine (SVM) and naïve Bayes (NB) classifier are more effective than other algorithms. The classification of Twitter posts is also performed in [24], where the primary role is assigned to the K-nearest neighbors (k-NN) and SVM. Huq et al. [25] provides detailed SA of user opinions from Twitter and Facebook social networks using convolutional neural networks (CNN), recurrent neural networks (RNN), long short-term memory (LSTM) neural networks, and hybrid approaches. In [26], comments on controversial political discussions in German on YouTube were conducted. SA was performed with various word embeddings, ML algorithms, and RNN. Then the classification efficiency was assessed using the following metrics: Precision, Recall, and F1-score. A new and more advanced approach to text classification using one CNN and two LSTM layers was described in [27].

All these works were mainly devoted to the analysis of texts in the English language. However, most texts and user comments are written in the Russian and Kazakh languages in the Kazakh media space. Thus, it became necessary to analyze the works dealing with these languages specifically. The sentiment classification of Russian tweets using logistic regression (LR), XGBoost, and CNN was carried out in [28, 29]. Unfortunately, the works devoted to the SA of Kazakh texts are greatly underrepresented. The Kazakh language is an agglutinative language with complex morphological and syntactic structures [30]. The sentiment classification tasks require the preprocessing stage, where stemmers or lemmatizers are applied to words to extract their stems or indefinite forms. The existing language packages of NLP tools do not contain the stemmers and lemmatizers for the Kazakh language as for other widely represented languages, especially European. Tukeyev et al., Yergesh et al. and Bekmanova et al. [31–33] implemented only a dictionary approach formalizing rules for defining the sentiment of phrases in texts. ML and NN approaches had a limited reflection in these works. In addition, they neither described any open-source analytics platforms nor provided functionalities for evaluating society’s SA and social mood in the Russian and Kazakh media spaces. Thus, various foreign and Kazakh analytics platforms were thoroughly investigated in the next section.

## Analytics platforms

The widespread development of Internet technologies, social networks [34], and data analytics has led to numerous tools and analytics platforms for promoting the brand, monitoring public opinions, and assessing social well-being as one of the main tools for determining the socio-economic system in the context of sustainable advancement.

Currently, the foreign market is represented by many tools for monitoring social networks [35], content analysis, and brand promotion. Therefore, the marketers distinguished a list of the most popular and advanced analytics platforms: Sproutsocial, Hubspot, Buzzsumo, Hootsuite, Brandmention, IQBuzz, and Snaplytics take an essential place.

Sproutsocial [36] is a multifunctional analytics tool that allows comparing results in several networks efficiently. This tool monitors and gathers all messages from Facebook, Twitter, Instagram, and other social networks in one unified place. It also benchmarks customer satisfaction by gaining analytics data through an automated Twitter DM survey. Sproutsocial is powered by ML algorithms that allow suggesting replies to users’ frequently asked questions. Generally, Sproutsocial is very useful when it is required to count links on Twitter [37], measure the growth of Instagram followers, evaluate participation on LinkedIn, and much more. This tool then provides an opportunity to evaluate results using understandable visualized reports. Sproutsocial includes marketing, social media management, and analytics of various leading brands and agencies, including Chipotle, Subaru, Zendesk, etc.

Hubspot [38] is a tool that allows marketers to obtain comparative information about the level of engagement on social networks and reflect on past efforts made to support high customer interest in their products. HubSpot provides a detailed overview of how social media affects profit margins and enables you to report on collected data quickly and efficiently. At the same time, it gives an opportunity to compare different platforms, track and view brands on social networks, and understand how the target audience watches business content. This tool has a bunch of features, such as website activity tracking, task management, insight, KPI dashboard, sales automation, etc. Website tracking saves how users interact with websites: visited pages, time spent on each page, the location of the visitor, and so on. This HubSpot feature allows businesses to track how a lead interacts with their website. The task management tool creates to-do lists and sets tasks’ priorities, statuses, and deadlines. The insight allows to automatically add the information about the company that was added to the application. This information includes the size of the company, its description, contact information, etc. The KPI dashboard sets the company’s goal for the sales and the performance of marketing planes. The sales automation feature has automated various stages of sales and deals. Another essential feature of the HubSpot analytics tool is the ability to analyze indicators specific to social networks and the entire path of the client. This tool also provides information about marketing tactics that are most effective for businesses and their impact on social media campaigns and includes dozens of other features for business.

BuzzSumo [39] is an excellent resource for analyzing the social interaction of any particular content. The tool allows searching for information based on requests on the Internet, taking into account various factors, including likes and reposts. The advanced search engine of BuzzSumo finds the most relevant content by topic, author, and domain. The service prompts which directions respond to the initially selected audience. Trying to choose the most accurate direction of content creation, it receives valuable information about answers on social networks. In addition, this tool allows collecting statistics on the number of reposts of a certain message on a blog on such social networks as Facebook [40], Twitter, and Pinterest. The main functionalities features of this platform are Content discovery (browsing topics, trends, and forums), Content research (crawling websites to get the most up-to-date content), Monitoring (finding different competitors, brand mentions, and updates and alerting with the most important events), and APIs (connect, integrate and develop with different sources of data). An essential feature of the tool is the ability to track the effectiveness of competitors as part of a content marketing campaign. BuzzSumo also easily determines competitors’ activity in social networks and identifies key people in a particular area. Such an analysis can help to see which posts receive the most engagement and use this data to adjust the content strategy. 

Hootsuite [41] is one of the most popular multifunctional services for working on social networks. The emphasis in this service is on working with Twitter, and, first of all, Hootsuite will be useful for those who maintain several accounts at once. Hootsuite also works successfully with Facebook, LinkedIn, MySpace, and Foursquare accounts and blogs on WordPress. HootSuite offers a wide range of analytical capabilities, such as connecting Google Analytics on the site and viewing graphs for comparing the number of tweets and the popularity of links. The key features of this platform are Post Scheduling, Streams, Analytics, and Assignments. Post Scheduling allows setting the dates and times to create a new post. Streams monitor active social media channels online. Analytics provide opportunities to see the performance of posts, their sentiments, page content clicks, total clicks on posts, and much more. Finally, assignments provide an ability to assign items to different team members. Hootsuite additionally allows you to post on all social networks on a specific schedule. The tool also allows you to track recent social trends and brand mentions.

Brandmention [42] is one of the most powerful platforms for free search and analysis of social networks. The system also offers SA, related keywords, popular sources, etc. Brandmention searches over 100 social networks [43], including social bookmarks, blogs, forums, social services, and more. In addition, data can be exported or configured for e-mail. Brandmention allows configuring the keywords for social monitoring and finding the company’s and its competitors' companies’ social handles. Some keywords can also be excluded from the search result.

IQBuzz [44] is a professional tool for analyzing and managing reputation on the Internet and a social network monitoring service [45]. IQBuzz tracks many sources and platforms such as Twitter, Yandex, LiveInternet, LiveJournal, various blogs, video hosting services such as RuTube and YouTube, various news, entertainment, and specialized services, and thematic and regional portals. One of the key advantages of the service is the ability to connect new sources and Internet resources for monitoring.

Snaplytics [46] is a cloud-based platform that analyzes Snapchat and Instagram stories. Today, millions of active Snapchat and Instagram users present stories as an excellent method of promotion on Instagram. This application also allows you to see peaks and slumps of views. The most important features of Snaplytics are automatic publishing, post scheduling, monitoring, and analytics. Platform users can track comments and replies, post stories from various sources, and view rates. Snaplytics also allows generating reports and exporting them to CSV files and other formats.

In Kazakhstan, social analytics is significantly underrepresented. Only a few works devoted to SA of the Kazakh language could be found in the Scopus database. Their research is mostly restricted to SA with the use of dictionary and ML approaches [30, 32]. Generally, there are only a few brands and social analytics platforms. Among the most advanced applications are the iMAS [47] and the Alem Media Monitoring [48], which work with the Russian and Kazakh languages. The iMAS platform provides SA on specified topics for a given period. The Alem Media Monitoring is software designed to analyze public opinion in the Internet space. This system allows collecting information on certain topics from news portals and social networks [49], determining the sentiment of texts using ML algorithms, visualizing all the performed analyses, and compiling and uploading reports. Unfortunately, these platforms are not open-source, and the information provided on their official websites demonstrates the study by three sentiment classes (positive, negative, and neutral) of texts and comments, the sources (news portals, social networks, and blogs), and periods of monitoring, visualizing them with different graphics and making reports in the word, excel and pdf formats. Nevertheless, there is no description of how these systems estimate the public’s social mood. Moreover, the research papers devoted to the iMAS and the Alem Media Monitoring platforms have not been found online. The proposed OMSystem was first described in [50]. It is designed to provide complex social analytics, including the web crawler, SA with sentiment dictionaries and ML algorithms, and evaluation of the “social well-being.” The following sections demonstrate the structure, functionalities, and module for evaluating the social mood of society.

## The OMSystem information system design methodology

The OMSystem, the first automatic tool developed to analyze the opinions of Kazakhstani users expressed through news portals, blogs, and social networks, was developed to provide a complex analysis of the public’s social mood and cover the parts skipped in other analytics platforms in Kazakhstan. The OMSystem allows monitoring of web resources and social networks with subsystems for modeling “social well-being” [51] and supporting sentiment dictionaries of the Russian and Kazakh languages and ML algorithms for determining the sentiment of texts and user comments. The OMSystem supports Kazakhstan’s leading news portals and popular social networks like Facebook, VKontakte, Instagram, Twitter, and YouTube. The platform’s main task is the operational monitoring of the information space and social networks on the most important topics in society. They unambiguously determine the scale of the problem, public opinion, and their quick explanation, analyze the dynamics of the commercial brand, events, and references to activities, and, in turn, assess the degree of “social well-being.”

This system allows working with texts in the Kazakh and Russian languages. It also has built-in modules for connecting to the application programming interfaces (APIs) of social networks: Vkontakte [52], Facebook [53, 54], Twitter [22, 55], Instagram [56, 57], YouTube [58], Telegram [59], and Odnoklassniki [60]. The OMSystem automatically determines the language of the text (Russian, Kazakh) and the sentiment of the topic, as negative, positive, or neutral, using a sentiment dictionary and ML algorithms. Furthermore, there is a possibility to record the time range in the system when monitoring social networks (for a year, for 6 months, for 3 months, for a month, for a week, for a day, etc.). The OMSystem also allows building visual reports on the monitoring results in various graphs and charts (pie, histogram, chart, graph, and others). At the same time, the platform provides ways to identify the profile of a social network participant by reading profile data and counting the activity of a participant in a topic by the number of comments, likes, and reposts.

The development of the OMSystem included the most important stages to achieve all the required goals. First, a module for using API to connect to social networks and a storage system for keeping the parsed data and processed analytical results were created. Then the sentiment dictionaries in the Russian and Kazakh languages were designed to evaluate the sentiment on the analyzed topics. The SA module was further extended with ML modules trained on the texts, labeled by human annotators and sentiment dictionaries. As an analytical application, the convenient quantitative and qualitative graphical visualization of the monitoring results was a significant step in the system’s design. The advanced role policy was the next important step. Finally, the system’s interface and design were improved to match the modern trends and requirements of the development of web applications.

The OMSystem was developed on the Django framework that uses the Python programming language. In addition, Django has its integrated authorization and authentical modules and libraries for web forms with input data validation. The administrative and parsed textual data is kept in the PostgreSQL relation database that is easily connected to the Django application. The SA modules with sentiment dictionaries and ML algorithms are shown in detail in the following chapters. The OMSystem has several roles: “Superuser,” “Administrator,” “User,” and “Expert.” The “Superuser” has the right to login into the System, navigate through the site, set up research and analysis reports, set up a rule profile for the search topic, change settings for uploading data from the System, invite experts, and view, edit, and delete personal data. The “Administrator” has the right to login into the System, view and edit system settings, assign roles for other users, change settings for connecting subsystems and modules, get technical reports (the number of results, the volume of data, search time, etc.), and configure settings for uploading reports. The “User” has the right to login into the System, navigate through the system, set up new topics and parameters for monitoring, and view the monitoring reports. The “Expert” has the right to login into the System, view the analysis page and details, switch to the sources of results, and view the system's functionality. JavaScript libraries and CSS styles were utilized to improve the interface of the application and graphical analytical reports.

**Fig. 1:**
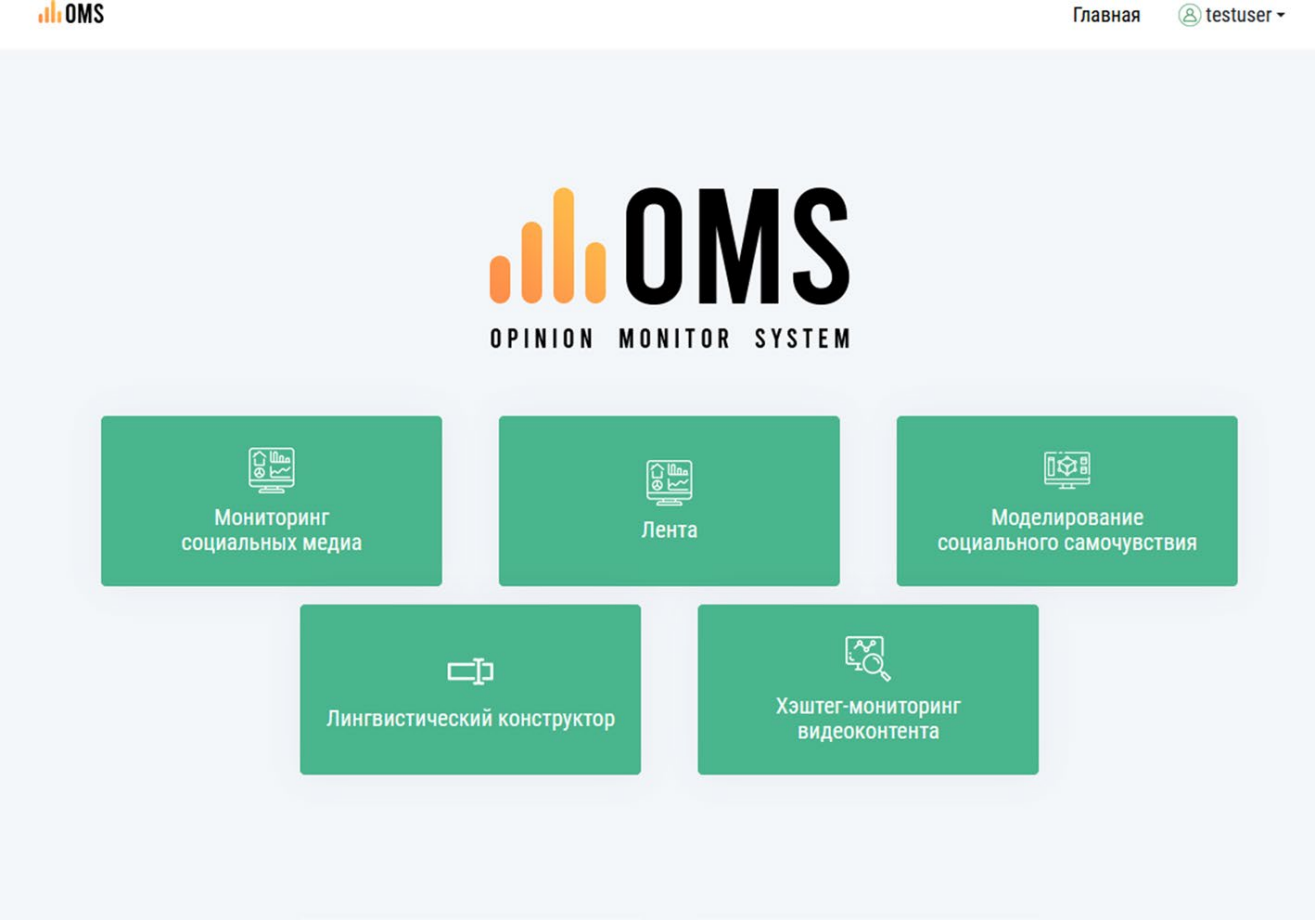
OMSystem’s interface

**Fig. 2:**
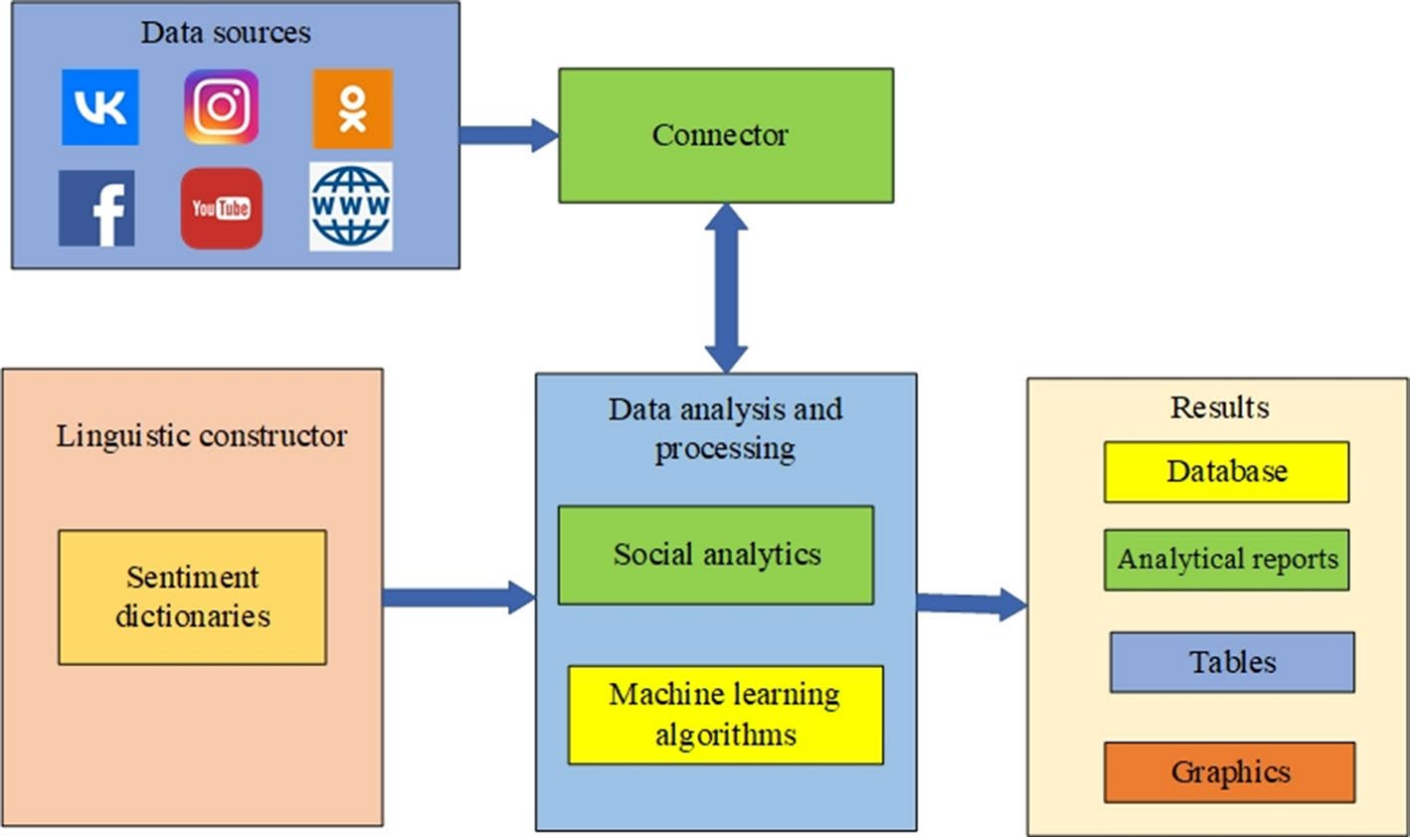
OMSystem’s architecture

The OMSystem’s interface and architecture are schematically shown in Figs. 1 and 2.

The English language is yet to be added to the interface of the platform. Its architecture was also described in [50], where experiments characterized the building of ML models for the OMSystem. The designed system’s functionality is implemented in the components:

- Data sources: They are represented by news portals, blogs, and social networks.
- Connector module: It is used for the connection to data sources.
- The linguistic constructor module: It is used for creating sentiment dictionaries that include words belonging to any of the three classes: positive, negative, and neutral.
- Data analysis and processing module: It uses sentiment dictionaries and ML algorithms for SA. In addition, this module creates social analytics defining social mood.
- Results module: It contains a formed relational database of texts and comments, analytical reports, graphics, and tables.

The SA tool, labeling texts and user comments in three sentiment classes (positive, neutral, and negative), is the core part of the OMSystem. The sentiment classes are assigned with the use of the hybrid approach: the lexicon-based (sentiment dictionaries) and the ML-based. The lexicon-based approach assigns a label by the largest number of words of one of three sentiment classes. The ML-based approach uses the trained ML models with the highest effectiveness in terms of accuracy, precision, recall, and F1-score, such as NB, LR, SVM, k-NN, Decision tree (DT), Random Forest (RF), and XGBoost.

## The linguistic module

A sentiment dictionary is generally represented as a list of words, each of which is assigned a “weight” that describes its emotional coloring. Sentiment dictionaries include hundreds or thousands of such words, and they are then used to determine the sentiment of sentences, paragraphs, or the whole texts based on the average of their weights of the sentiment words. The sentiment dictionaries in the OMSystem are also directed to analyzing social, political, and economic content, so they need to include corresponding words for such texts.

In the OMSystem, the sentiment dictionaries were developed in the following steps:

1. Forming a sentiment vocabulary, which is marked on the basis of feelings and emotions. The sentiment dictionary consists of such elements as words, phrases, misspelled words and slang forms of words, each of which has its own emotional aspect.
2. Creating words with errors in Russian and Kazakh languages, which will increase the search results. The words with errors are formed by replacing, inserting, and deleting symbols.
3. Filling the dictionary. The dictionary is based on a sentiment dictionary of English words from open sources, categorized by their sentiment (https://public.tableau.com/views/NRC-Emotion-Lexicon-viz1/NRCEmotionLexicon-viz1?:embed=y&:toolbar=yes&:loadOrderID=0&:display_count=yes&:showTabs=y&:tabs=no&:showVizHome=no). It is stated that this dictionary is suitable for any language, so the words from this dictionary were translated into Russian and Kazakh.
4. Expert linguists were involved in labeling the sentiment of words of the newly parsed news topics and social media comments to increase the size of the sentiment dictionaries and fill them with new important words.

Currently, the Russian sentiment dictionary includes 44,381 words, and the Kazakh sentiment dictionary includes 29,654 words.

The linguistic module defines the sentiment of texts with the use of the formed sentiment dictionaries. Here is used a function that calculates the sentiment by the maximum number of positive, negative, and neutral words in the text. This approach’s effectiveness greatly depends on the quality of the designed sentiment dictionary [61]. Although this approach is very effective, creating a high-quality sentiment dictionary requires much effort. After an initial sentiment dictionary is created manually, it is then expanded by the synonyms and antonyms from larger dictionaries existing for many languages.

In the OMSystem, large sentiment dictionaries for the Russian and Kazakh languages are developed. The following formula finds the sentiment of the text:

1 $$S_{t} = \left\langle {Max(w_{pos} ,w_{neut} ,w_{neg} ),D} \right\rangle ,$$

where $S_{t}$ is a sentiment of the text; $w_{pos}$ is the number of positive words; $w_{neut}$ is the number of neutral words; $w_{neg}$ is the number of negative words; $D$ is a sentiment dictionary.

**Fig. 3:**
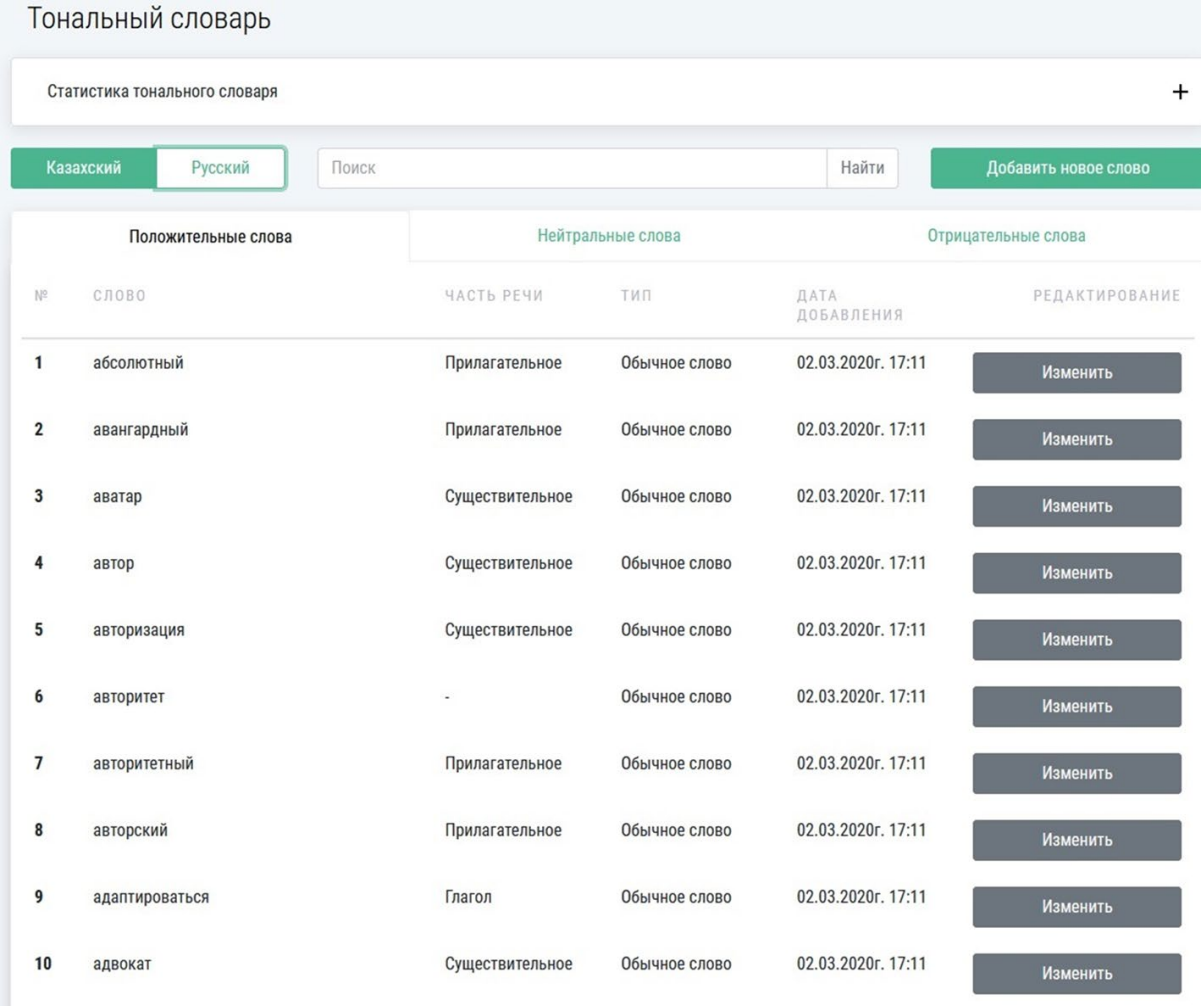
The Russian sentiment dictionary

**Fig. 4:**
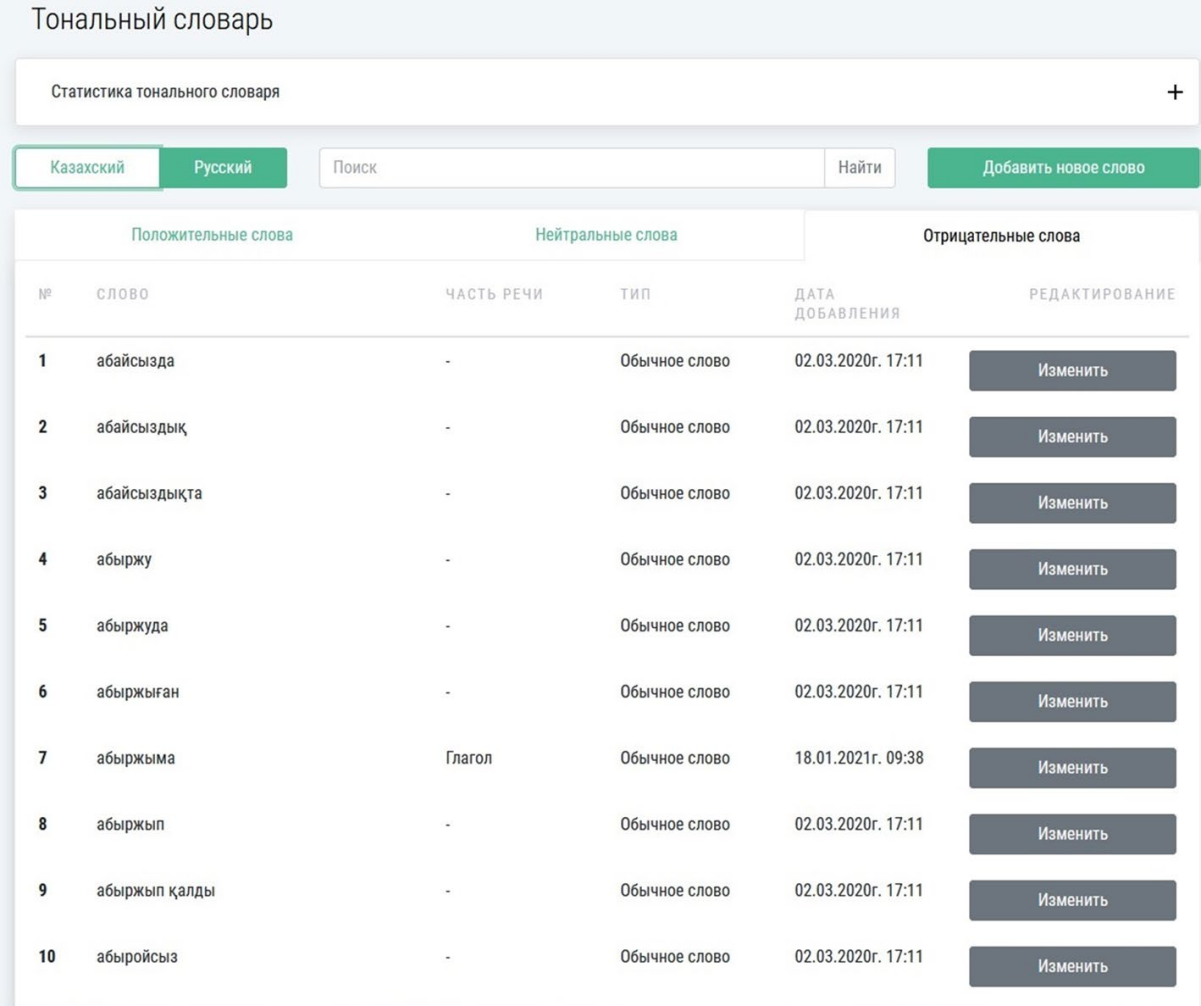
The Kazakh sentiment dictionary

The sentiment dictionaries of both languages used in the OMSystem are presented in Figs. 3 and 4.

## Machine learning methods

In addition to sentiment dictionaries, ML algorithms are also used in the OMSystem to label the text data. The following algorithms are implemented in the system: NB, LR, SVM, k-NN, DT, RF, and XGBoost. The model for defining sentiment with ML algorithms is calculated by the formula:

2 $$S_{t} = \left\langle {M,T} \right\rangle ,$$

where $S_{t}$ is the sentiment of a text; $M$ is an ML model; $T$ is a text document.

An NB classifier [62] is one of the simplest and most commonly used ML algorithms for text classification that uses a probabilistic approach based on the Bayes theorem with strong data independence assumptions. It considers every feature that affects the probability, regardless of the presence or absence of any other features. In text classification, NB is trained on documents for each class, where the conditional probability that document $d$ belongs to class $c$ is computed. This formula is represented by the expression:

3 $$P(c|d) = \frac{P(c) \times P(d|c)}{{P(d)}},$$

where $d = \{ x_{1} ,x_{2} , \ldots ,x_{n} \}$, $x_{i}$ is a weight of the $i{\text{th}}$ word in a document $d$, and $c$ is a class of the document.

SVM [63] is another popular ML algorithm. This algorithm works with the feature space separated by hyperplanes. In this case, a good separation is achieved due to the hyperplane, which has the greatest distance to the nearest points of the training data of the two classes (the so-called functional boundary), since the larger the boundary, the lower the classifier error. The formula of SVM is given below:

4 $$y_{i} (\overrightarrow {w} \times \overrightarrow {x} + b) \ge 0,$$

where $\overrightarrow {x} = (x_{1} , x_{2} , \ldots , x_{n} )$ is a feature vector; $\overrightarrow {w} = (w_{1} ,w_{2} , \ldots ,w_{n} )$ is a weight vector;$y_{i}$ are output values; $b$ is a bias.

If the value is greater than or equal to zero, it belongs to the positive class. Otherwise, it is in the negative class.

**Fig. 5:**
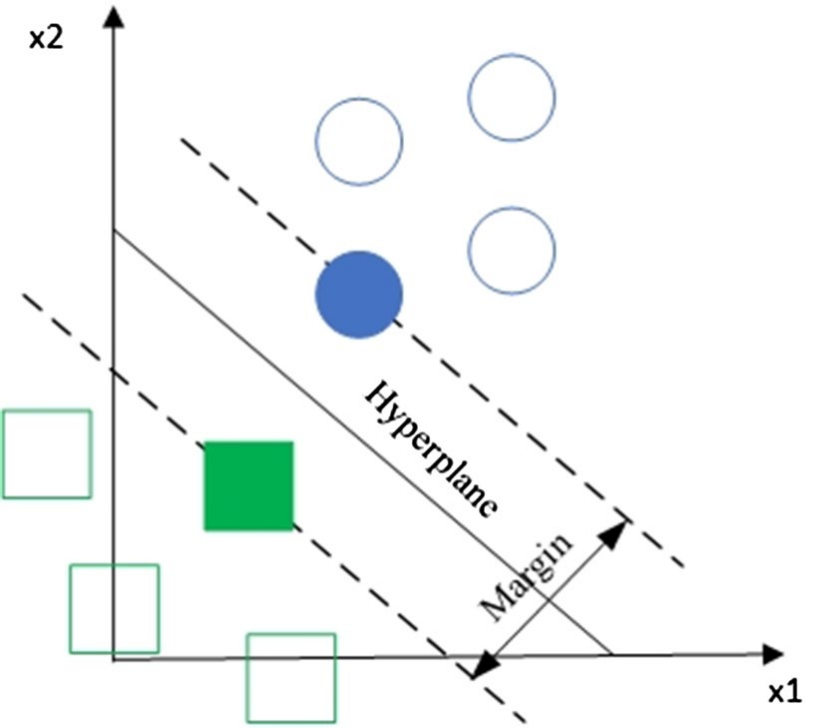
A hyperplane of an SVM

A splitting hyperplane of SVM mainly works with two-class classifiers. However, it can easily be adapted to multiclass classification, using a set of “One-vs-All” classifiers. A hyperplane of SVM is shown in Fig. 5.

**Fig. 6:**
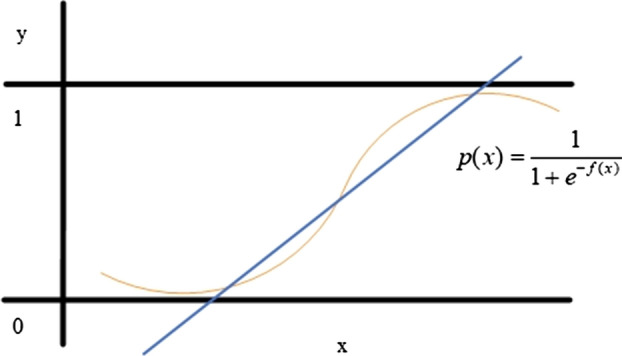
A logistic function

An LR classifier [64] predicts the probability of an independent variable in the interval [0,…,1] using a logistic function:

5 $$p(x) = \frac{1}{{1 + e^{ - f(x)} }},$$

where $f(x) = w_{0} + w_{1} x_{1} + \cdots + w_{r} x_{r}$ is a linear classification function; $\overrightarrow {x} = (x_{1} ,x_{2} , \ldots ,x_{n} )$ is a feature vector; $\overrightarrow {w} = (w_{1} ,w_{2} , \ldots ,w_{n} )$ is a weight vector. A logistic function $p(x)$ is presented as a sigmoid with the values of probability of 0 and 1. Document $d$ belongs to class 1 if the value $p(x)$ moves to 0. Otherwise, it is put into class 2. In the case of multiclass classification, a “One-vs-All” and “One-vs-One” approaches are used to identify a specific class. A logistic function is shown in Fig. 6.

A k-NN algorithm [65] is one of the simplest data classification algorithms. It calculates distances between vectors and assigns points to the class of its $k$ nearest neighbor points. This algorithm usually classifies documents using the most widely used distance measure called Euclidean distance, which is defined as:

6 $$d(x,y) = \sqrt {\sum\nolimits_{i = 1}^{N} {(a_{ix} - a_{iy} )^{2} } } ,$$

where $d(x,y)$ is a distance between 2 documents; $a_{ix}$ and $a_{iy}$ are the weights of the $i{\text{th}}$ term in documents $x$ and $y$, correspondingly; $N$ is the number of a unique word in a set of documents. This algorithm plainly memorizes all feature vectors and their corresponding class labels during the training stage. When working with real data, the unknown class labels, the distance between the new observation vector and the previously stored ones is calculated. Then the $k$ nearest vectors are selected, and the new object belongs to the class to which most of them belong.

**Fig. 7:**
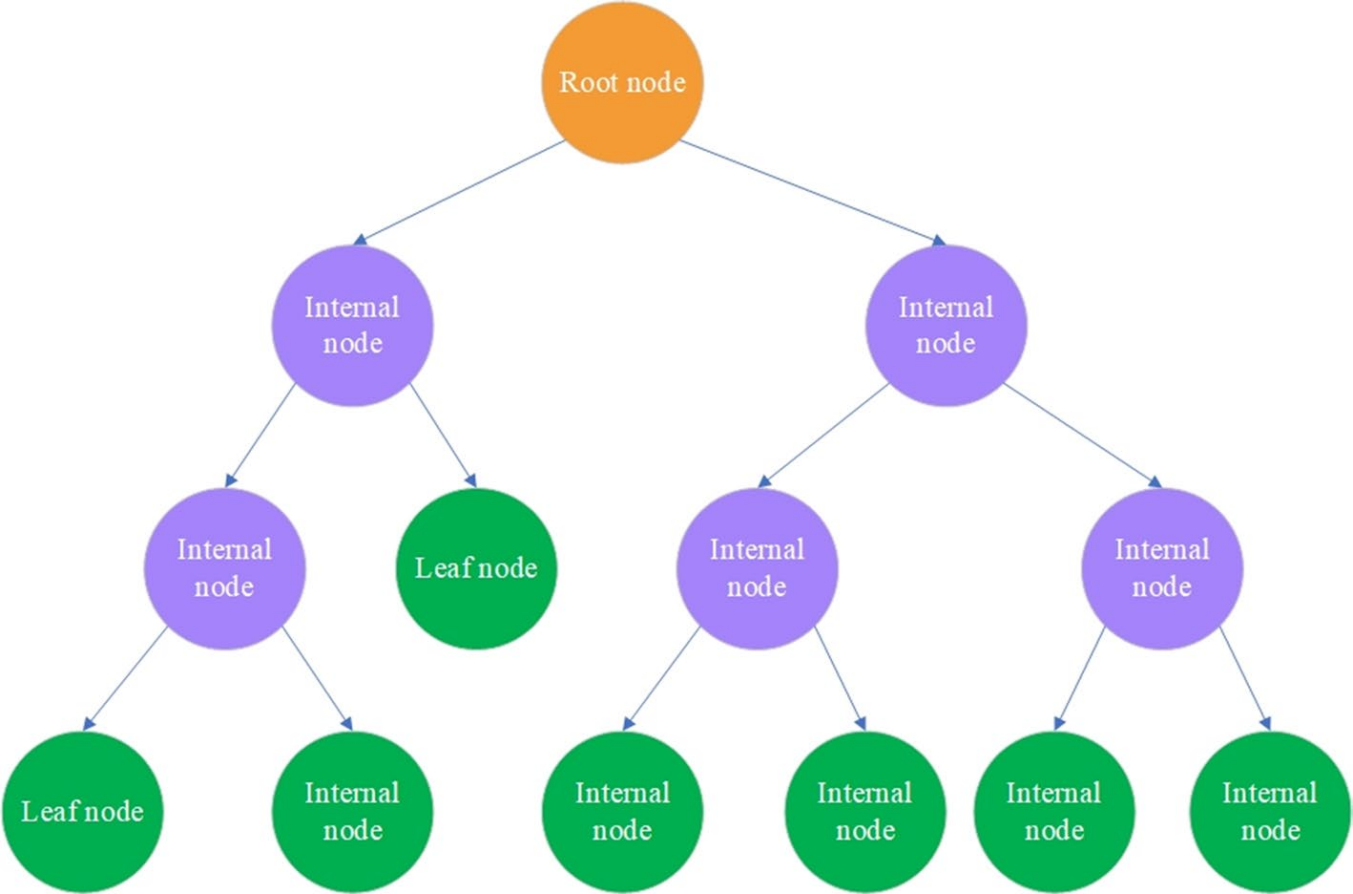
Decision tree

DT [66] is a supervised learning method that uses a set of rules to make decisions the same way a person makes decisions. This method divides a data set by features and answers specific questions until all data points belong to a particular class. Thus, a tree structure is formed by adding a node for each question. The first node is the root node. At the first classification step, a word is selected, and all documents containing it are placed on one side, and documents that do not contain it are put on the other side. As a result, two sets of data are obtained. Then a new word is selected in these sets, and all previous steps are repeated. The same procedure continues until the entire dataset is partitioned and assigned to leaf nodes. If all data points in a leaf node uniquely correspond to the same class, then the class of the node is well-defined. In the case of mixed nodes, the algorithm assigns the given node the class with the largest number of related data points. DT is shown in Fig. 7.

**Fig. 8:**
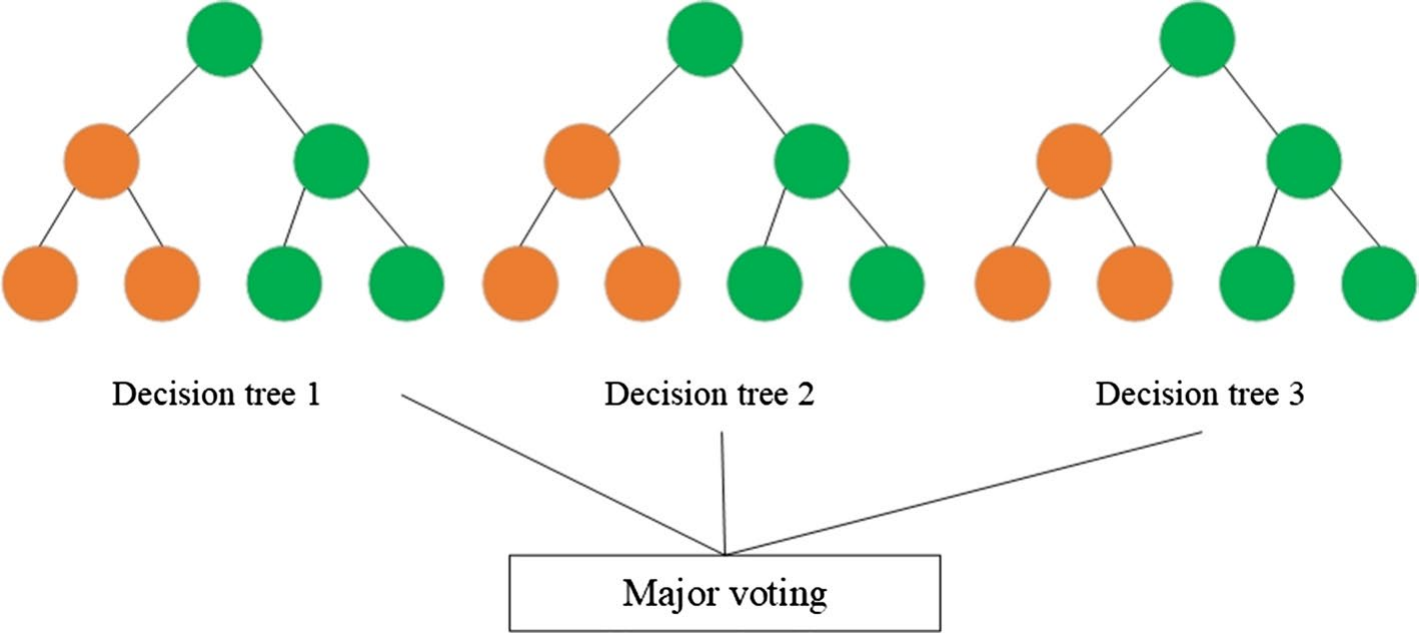
Random forest

RF [67] is another popular ML algorithm based on the concept of ensemble learning. This concept involves combining multiple classifiers to improve model performance. This algorithm includes not a single DT but a bunch of them. In classification problems, each document is classified by all trees independently. At the output, the class of the document is determined by the largest number of votes among all trees. RF is shown in Fig. 8.

XGBoost [68] is considered one of the most superior and advanced methods among all ML algorithms, which uses the principle of boosting. This method also implements an ensemble technique as an RF algorithm. The deviations of the trained ensemble predictions are computed on the training set at each iteration. Thus, optimization is performed by adding new tree predictions to the ensemble, reducing the mean deviation of the model. In addition, XGBoost allows tuning many different hyperparameters to increase the model’s performance.

## Data collection and data processing

**Fig. 9:**
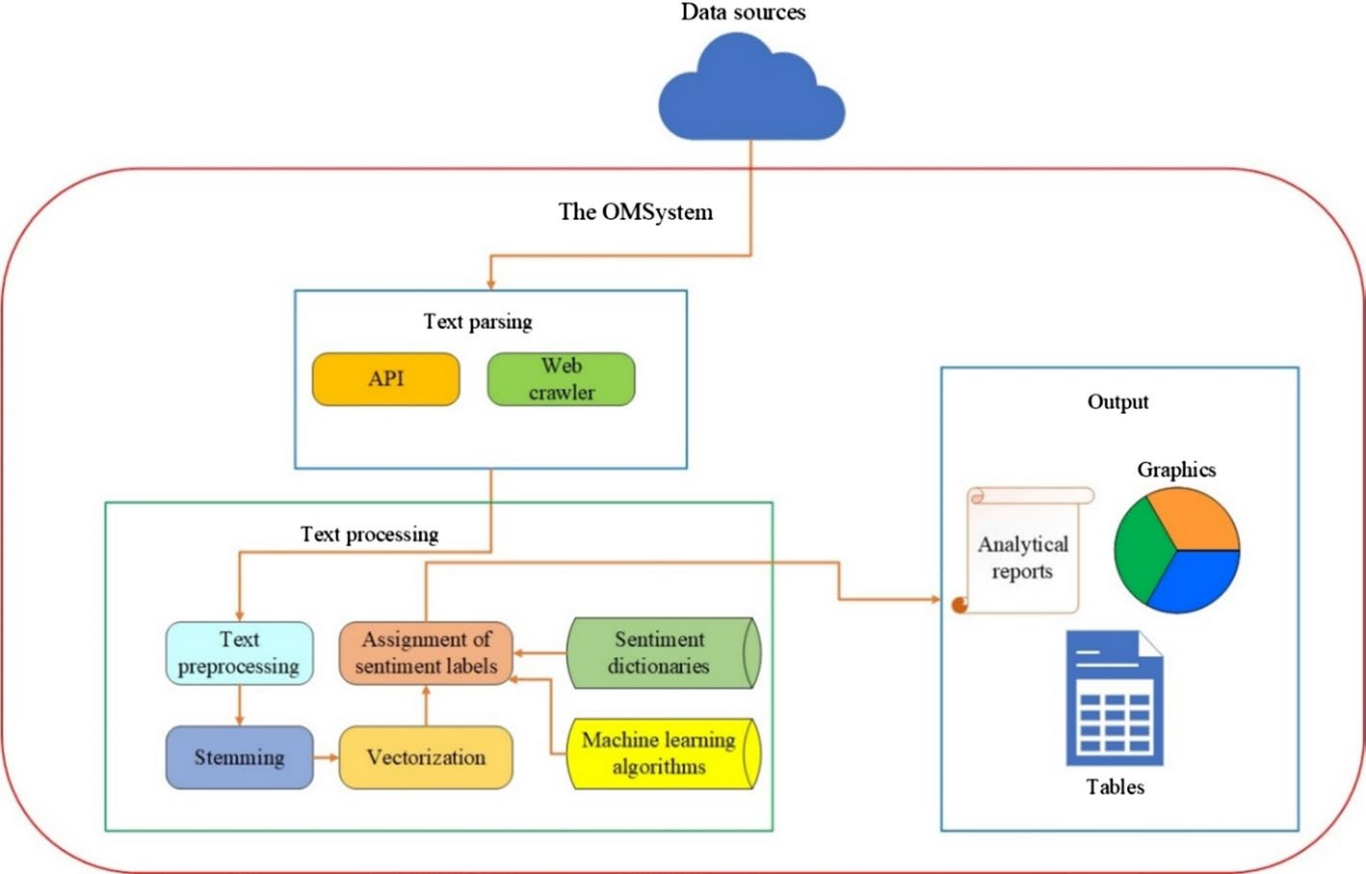
The OMSystem’s analytics building steps

The web-crawler of the OMSystem parses texts and user comments from different sources, such as Kazakhstan’s news portals, social networks, and blogs. The parsed texts are aggregated in the designated PostgreSQL database. The scheme of the OMSystem’s functioning is presented in Fig. 9.

After the texts are gathered in the database, it is required to apply the following steps before training ML models:

- Text preprocessing
- Stemming
- Vectorization
- Class resampling

These mentioned steps are thoroughly described in the following sub-sections.

### Text preprocessing and stemming

All words are converted to lower case at the preprocessing stage, and extra words, symbols, punctuation marks, and links are removed. Then it is also necessary to remove the stop words, which are words that do not carry much semantic content. Examples of such words are prepositions, conjunctions, pronouns, etc. (“нa”—“on,” “в”—“in,” “бәpi”—“all,” “жәнe”—“and,” “бipaқ”—“but” and others). Another important step is methods for reducing the number of words with similar meanings. These methods are called stemming and lemmatization. In stemming, affixes and endings of words are removed to obtain their stems. In lemmatization, words are reduced to their indefinite forms. Stemming is an easier way to write an algorithm for removing parts of words. Lemmatization, on the contrary, requires significant efforts to develop rules for reducing words to the infinitive form. The NLTK Python library includes excellent stemmers for the Russian and English languages. Unfortunately, it does not yet contain the same well-developed stemmer for the Kazakh language. Thus, a new stemmer called “KazakhStemmer” has been developed for getting stems of the Kazakh words.

### Vectorization

After text preprocessing, the vectorization stage is performed, where the Bag of words (BOW) and Term frequency-inverse document frequency (TF-IDF) [69] techniques are widely used. The BOW model is quite simple, and it is easy to use for feature extraction. The model’s simplicity lies in the fact that it does not take into account either the order, the structure of words, or the features present in it. The model only considers whether the known word occurs in the document or not. The dictionary of words comprises all the words found in all documents. For example, given a number of documents and their corresponding vector representations:

- I am writing—[1, 1, 1, 0, 0, 0, 0, 0]
- I am writing a poem—[1, 1, 1, 1, 1, 0, 0, 0]
- I am writing a poem in the library—[1, 1, 1, 1, 1, 1, 1, 1]

Vectorization involves counting the number of words in each document. It is shown in Table 1.

**Table 1:** Vectorization with BOW

Documents	I	am	writing	a	poem	in	the	library
I am writing	1	1	1	0	0	0	0	0
I am writing a poem	1	1	1	1	1	0	0	0
I am writing a poem in the library	1	1	1	1	1	1	1	1

Despite its simplicity, the BOW algorithm has a significant drawback associated with an increase in the size of vectors in the case of a large number of documents. Then vectors contain many zeros. The TF-IDF metric is utilized to solve this problem. This metric is a statistical measure used to rate the importance of a word in the context of a document that is part of a document collection or corpus. The weight of a word is proportional to the number of occurrences in the document, and inversely proportional to the frequency of occurrence of the word in other documents in the collection. TF (Term frequency) is the ratio of the number of occurrences of a certain word to the total number of words in the document. Thus, the importance of a word $t_{i}$ within a single document is evaluated by the formula

7 $$tf(t,d) = \frac{{n_{i} }}{{\sum\limits_{i = 1}^{k} {n_{i} } }},$$

where $n_{i}$ is the number of occurrences of a word in the document, and the denominator is the total number of words in the document.

Inverse document frequency (IDF) is the inversion of the frequency with which a certain word occurs in the documents of the collection. Accounting for IDF reduces the weight of commonly used words. For each unique word within a given collection of documents, there is only one IDF value

8 $$idf(t,D) = \log \frac{|D|}{{|(d_{i} \supset t_{i} )|}},$$

where $|D|$ is the number of documents in the corpora; $|(d_{i} \supset t_{i} )|$ is the number of documents where $t_{i}$ occurs.

When both $TF$ and $IDF$ values are found, the two parts are multiplied

9 $$TF - IDF = TF \times IDF.$$

The texts in the following experimental part are vectorized with the $TF - IDF$ metric.

### Class resampling

During a training step of the classification model, a dataset often contains unequal classes. This case causes a significant problem when the most represented class labels most dataset elements. As a result, although accuracy is high, the values of precision, recall, and F1-score metrics remain low. Several approaches exist to resample classes: Random oversampling, Random undersampling, and Synthetic minority oversampling (SMOTE) [50].

**Fig. 10:**
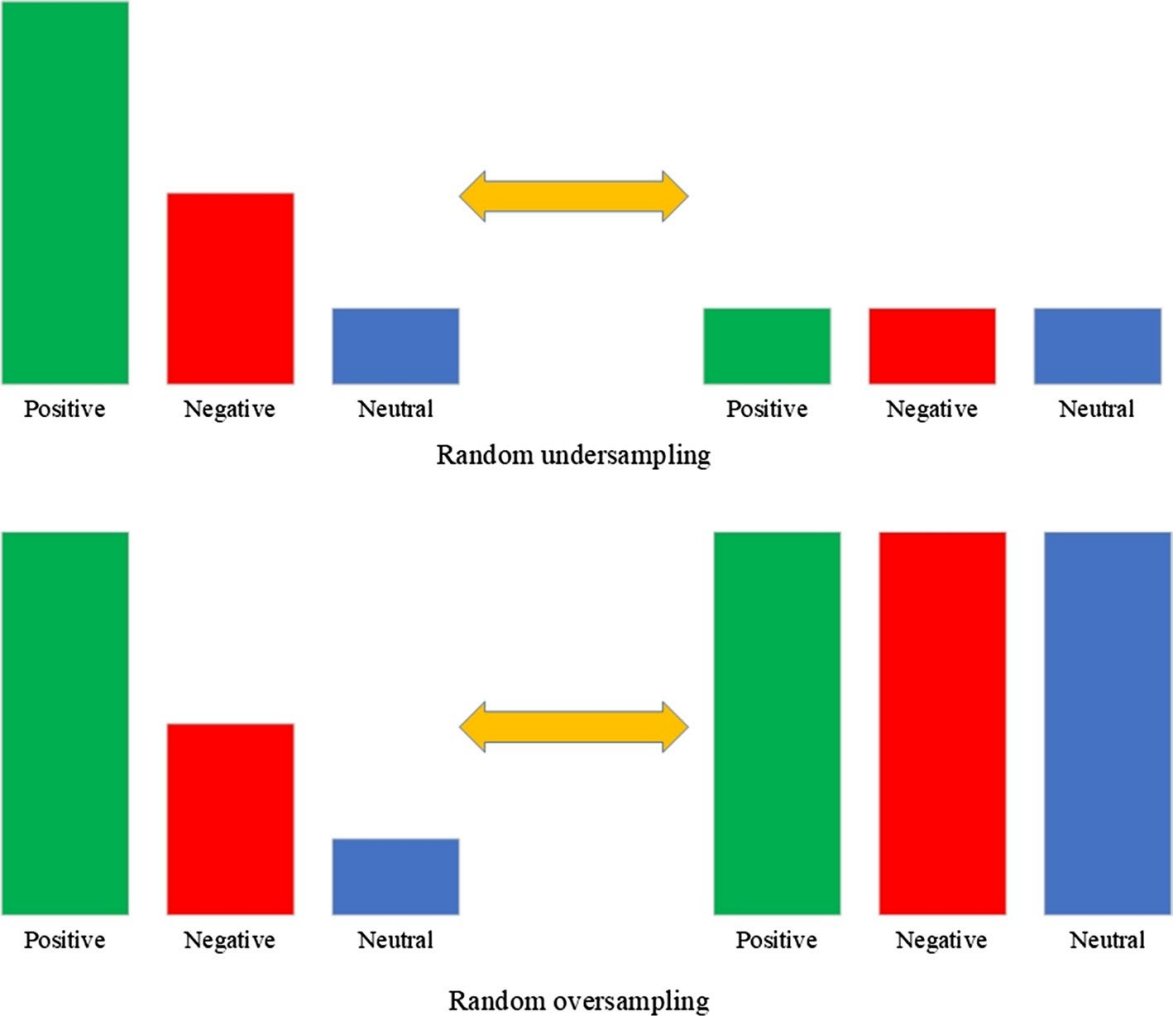
Random undersampling and oversampling techniques

**Fig. 11:**
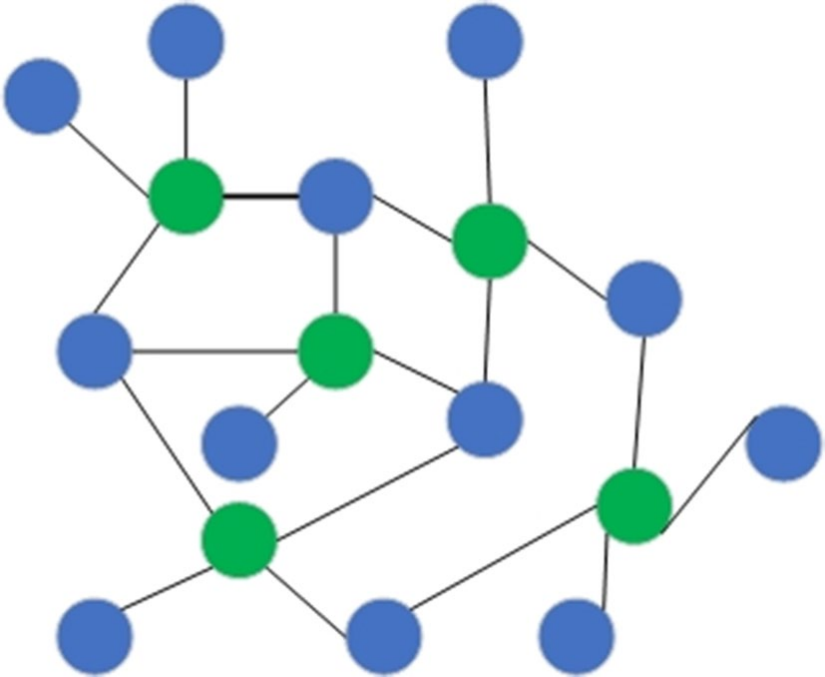
SMOTE

In Random undersampling, the sizes of the large classes are reduced to the smallest class to make them all equal. In Random oversampling, an opposite operation is done. Small classes are increased to the size of the most significant class. Even though these methods will equalize the classes, they have some drawbacks. Random undersampling eliminates a considerable portion of useful information in the classes, and the dataset is greatly decreased in size. Random oversampling saves valuable data but does not replenish new information, just copying the existing one several times. SMOTE is another method that effectively increases class sizes by creating new synthetic data points between existing elements. This procedure not only preserves important information but supplements it with new data. The class resampling techniques are shown in Figs. 10 and 11.

### Multiclass classification metrics

After texts are preprocessed, vectorized, and balanced, they are classified with ML algorithms. In order to evaluate the correctness and efficiency of the performance of classification, the following accuracy, precision, recall, and F1-score metrics are utilized [50]:

10 $$accuracy = \frac{TP + TN}{{TP + FP + TN + FN}},$$

11 $$precision = \frac{TP}{{TP + FP}},$$

12 $$recall = \frac{TP}{{TP + FN}},$$

13 $$F1\_score = 2\frac{precision \times recall}{{precision + recall}},$$

where *TP* (true positive) are elements that are truly classified with the *positive* sentiment class; *TN* (true negative) are elements that are truly classified with the *negative* sentiment class; *FP* (false positive) are elements that are falsely classified with the *positive* sentiment class; *FN* (false negative) are elements that are falsely classified with the *negative* sentiment class.

In multiclass classification, the stated metrics have to be transformed into accuracy, precision-macro, precision-micro, precision-weighted, recall-macro, recall-micro, recall-weighted, F1-score-macro, F1-score-micro, and F1-score-weighted. Precision-macro is the arithmetic mean of all class precision scores. Precision-micro is the sum of all true positives for all classes divided by all positive predictions

14 $$precision\_macro = \frac{{precision_{1} + precision_{2} + precision_{3} }}{3},$$

15 $$precision\_micro = \frac{{TP_{1} + TP_{2} + TP_{3} }}{{TP_{1} + TP_{2} + TP_{3} + FP_{1} + FP_{2} + FP_{3} }}.$$

Recall-macro and recall-micro are defined in a similar manner

16 $$recall\_macro = \frac{{recall_{1} + recall_{2} + recall_{3} }}{3},$$

17 $$recall\_micro = \frac{{TP_{1} + TP_{2} + TP_{3} }}{{TP_{1} + TP_{2} + TP_{3} + FN_{1} + FN_{2} + FN_{3} }}.$$

The weighted metrics are calculated in the same manner as macro metrics, but each class has its own weight depending on the number of elements that are in that class.

18 $$precision\_weighted = \frac{{w_{1} \times precision_{1} + w_{2} \times precision_{2} + w_{3} \times precision_{3} }}{3},$$

19 $$recall\_weighted = \frac{{w_{1} \times recall_{1} + w_{2} \times recall_{2} + w_{3} \times recall_{3} }}{3},$$

where $w_{1} ,w_{2} ,\,\,and\,w_{3}$ are the weights of the corresponding classes.

Accuracy, precision, recall, and F1-score metrics measure how well the data is classified. The metrics values have to be closer to 1 to show better performance. They are used in almost every research, where ML classification models are trained. The experimental part of this paper pays much attention to measuring the performance of the trained models with these metrics.

Another metric that shows the opposite tendency is Logarithmic Loss (LogLoss). This metric is calculated by the formula

20 $$LogLoss = - \frac{1}{N}\sum\limits_{i = 1}^{N} {\sum\limits_{j = 1}^{M} {y_{ij} *\log (p_{ij} )} } ,$$

where $y_{ij}$ shows whether an element $i$ belongs to a class $j$; $p_{ij}$ is the probability of an element $i$ belonging to a class $j$; $N$ is the total number of elements; $M$ is the total number of classes.

When a value of LogLoss is near 0, it shows the high accuracy of classification.

Furthermore, two metrics called Mean Absolute Error (MAE) and Mean Squared Error (MSE) are used to evaluate the performance of ML algorithms. MAE is the average of the difference between the original values and the predicted values. MSE differs from MAE in that it takes the average of the square of the difference between the original values and the predicted values. They are calculated by the formulas

21 $$MAE = \frac{1}{N}\sum\limits_{i = 1}^{N} {|y_{i} - \widehat{{y_{i} }}|} ,$$

22 $$MSE = \frac{1}{N}\sum\limits_{i = 1}^{N} {(y_{i} - \widehat{{y_{i} }})^{2} } ,$$

where $y_{i}$ is a predicted value of an element; $\widehat{{y_{i} }}$ is a real value of an element; $N$ is the total number of elements.

However, they are good for regression tasks, not classification tasks. Therefore, these metrics are not used in this research.

**Fig. 12:**
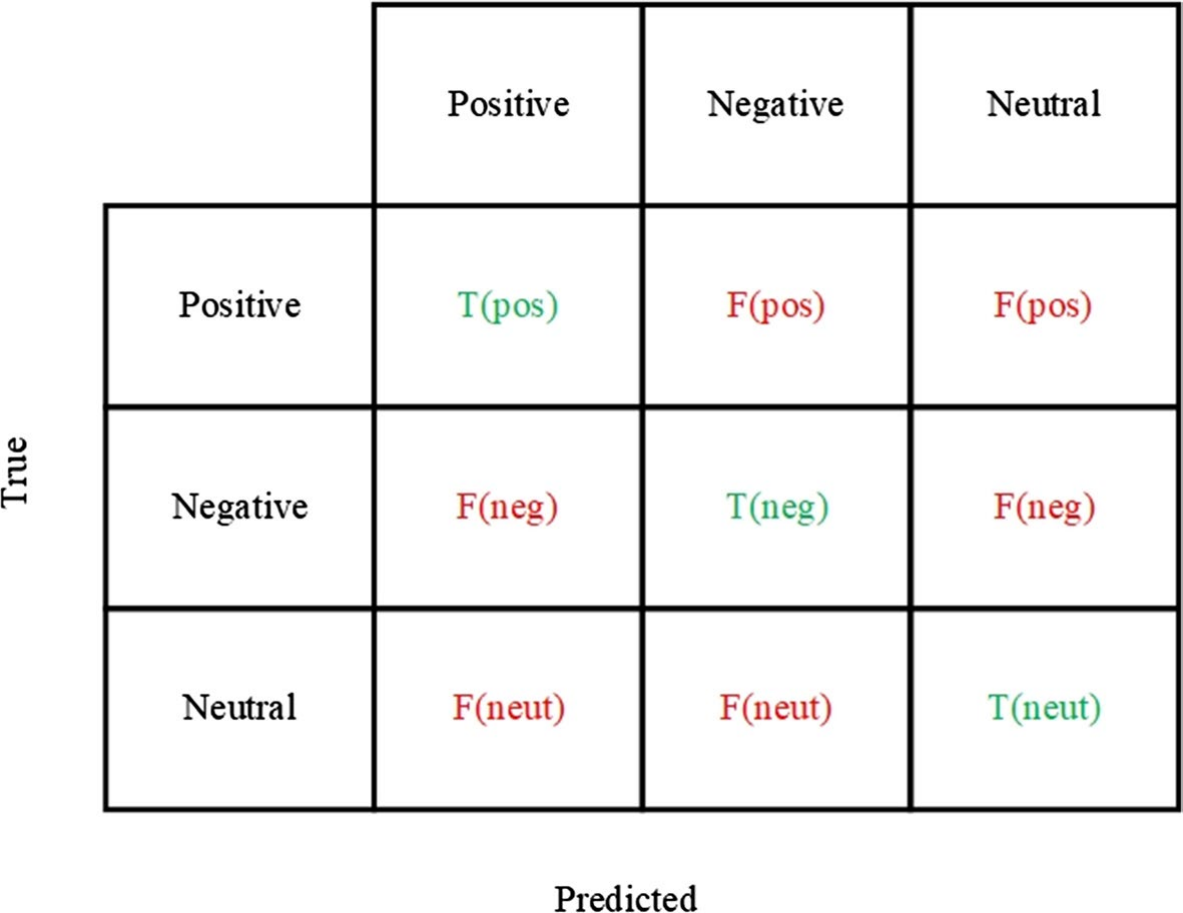
A confusion matrix

There are also useful graphical measures for effectively evaluating the algorithms. They are called a confusion matrix and Area Under Receiver Operating Characteristics. The confusion matrix shows true and false predictions for every class. In the multiclass classification, it is shown in Fig. 12. Area Under Receiver Operating Characteristics (AUC–ROC) is very convenient for visualizing classification results. It represents an area under the curve on the plane of axes in the range from zero to one. The axes of the planes show True positive rate and False positive rate, which are calculated by the following formulas

23 $$True\,Positive\,Rate = \frac{TP}{{TP + FN}},$$

24 $$False\,Positive\,Rate = \frac{FP}{{FP + TN}},$$

**Fig. 13:**
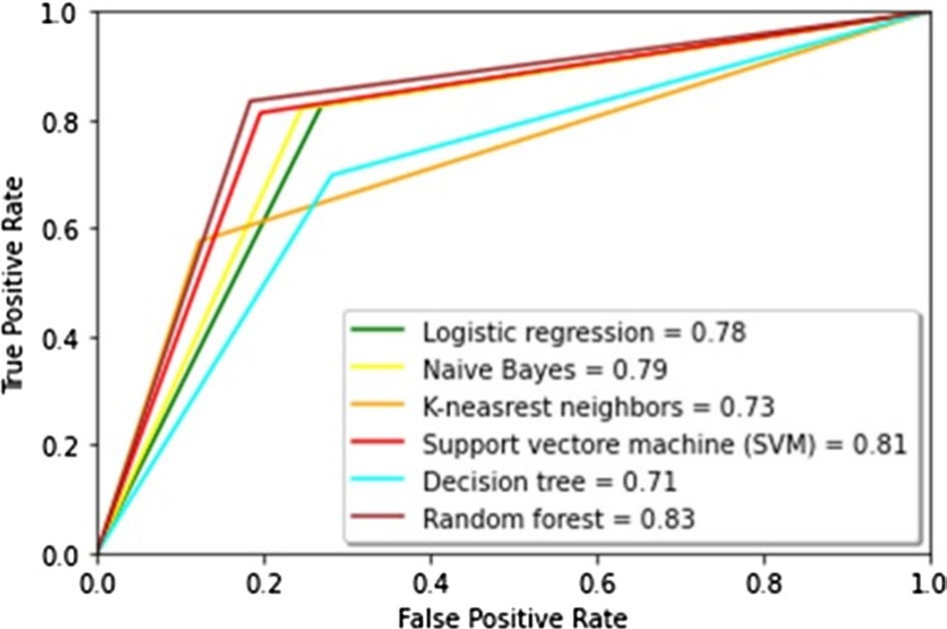
A plot of an AUC–ROC curve

The greater the value of an area, the better the classification model’s performance is. Although the AUC–ROC metric is a very important metric for evaluating the performance of models, it is standardly used for binary classification problems. In order to adapt it for multiclass classification, “One-vs-All” or “One-vs-One” techniques are utilized. An example of the AUC–ROC curve is shown in Fig. 13.

In the experiments conducted in Chapter 9, accuracy, precision, recall, and F1-score metrics were supplemented with confusion matrices and AUC–ROC curves to show the classification results.

## Defining the social mood of society

While OMSystem provides a comprehensive analysis of the texts of Kazakhstan Internet resources and reveals the sentiment of user opinions using ML methods, it also allows evaluating the semantic profile of society’s response to various events. The models of engagement assessment standards are considered to implement these steps. They are based on the method of measuring social network indicators for social media marketing management (SMMM) with the use of special SocialBakers formulas from Facebook [70]. The presented metrics are considered and adapted for social analytics. They are presented below:

- the level of interest in the topic in society ($R_{CT}$);
- the level of topic discussion activity in society ($R_{CE}$);
- the level of social mood ($R_{TS}$).

The level of interest in the topic $R_{CT}$ is calculated using the following formula:

25 $$R_{CT} = \frac{CT \times 100\% }{{\max_{CT} }},$$

where $CT$ is the number of texts or comments found on a particular topic. $\max_{CT}$ is the maximum number of texts or comments on a certain topic (set by the expert for a certain time). The range of values starts from 0% and is not bounded. If the value exceeds 100%, it means that this topic is of great interest.

$R_{CE}$ determines interaction in social networks and shows the level of topic discussion activity in society. This indicator allows assessing how differently the audience reacts to the categories of events in society. It is calculated using the formula:

26 $$R_{CE} = \frac{{\frac{L + R + C}{{CP}}}}{CS} \times 100\% ,$$

where $CS$ is the sum of the number of subscribers; $CP$ is the number of texts found on a certain topic; $C$ is the number of comments; $L$ is the number of likes; $R$ is the number of reposts. The range of values starts from 0% and is not bounded. As there are many topics on each news portal or a group in a social network and all users and subscribers cannot discuss them all, the level of topic discussion activity is usually not a big number.

$R_{TS}$ is the level of social mood, which is defined by the maximum value of the sums of positive, neutral, and negative texts or comments on a certain topic.

## Experimental part

### Developing ML models for the OMSystem

The first experiments are devoted to the development of ML algorithms for classifying textual data. The Python programming language is utilized to conduct these experiments on the Jupyter Notebook platform. The NLTK library is used for preprocessing and stemming the data. The Scikit-learn library vectorizes the data and contains ML algorithms for classification. The Imbalanced-learn library serves for resampling classes. Finally, Seaborn and Matplotlib visualize all the results. The datasets parsed by the OMSystem’s web crawler were distributed in the following way by the languages and sentiment classes (Table 2).

**Table 2:** Distribution of texts by classes

Language	Negative	Positive	Neutral
Russian	24,636	82,360	4919
Kazakh	1732	18,234	642

The datasets for the Russian and Kazakh languages have been preprocessed, vectorized with the *TF-IDF* metric, and resampled with the Random oversampling, Random undersampling, and SMOTE techniques. Then the datasets were randomly split into training and testing sets as 70% and 30%, respectively, and classified with NB, SVM, LR, k-NN, DT, RF, and XGBoost [71] ML algorithms. The results of the classification of imbalanced Russian and Kazakh datasets are shown in Tables 3 and 4.

**Table 3:** The classification metrics for the imbalanced Russian texts

Classifier	NB	SVM	LR	k-NN	DT	RF	XGBoost	Average
Accuracy	0.75	0.74	0.80	0.76	0.73	0.81	0.76	0.76
Precision-macro	0.80	0.25	0.78	0.62	0.58	0.79	0.72	0.65
Precision-micro	0.75	0.74	0.80	0.76	0.73	0.81	0.76	0.76
Precision-weighted	0.76	0.54	0.79	0.74	0.79	0.81	0.73	0.74
Recall-macro	0.39	0.33	0.52	0.44	0.60	0.57	0.42	0.47
Recall-micro	0.75	0.74	0.80	0.76	0.73	0.81	0.76	0.76
Recall-weighted	0.75	0.74	0.80	0.76	0.73	0.81	0.76	0.76
F1-score-macro	0.38	0.28	0.57	0.46	0.54	0.63	0.45	0.47
F1-score-micro	0.75	0.74	0.80	0.76	0.73	0.81	0.76	0.76
F1-score-weighted	0.67	0.63	0.78	0.70	0.75	0.79	0.70	0.72
Average	0.68	0.57	0.74	0.68	0.69	0.76	0.68

**Table 4:** The classification metrics for the imbalanced Kazakh texts

Classifier	NB	SVM	LR	k-NN	DT	RF	XGBoost	Average
Accuracy	0.89	0.89	0.89	0.89	0.87	0.91	0.89	0.89
Precision-macro	0.43	0.30	0.67	0.59	0.61	0.83	0.75	0.60
Precision-micro	0.89	0.89	0.89	0.89	0.87	0.91	0.89	0.89
Precision-weighted	0.82	0.79	0.87	0.87	0.90	0.91	0.88	0.86
Recall-macro	0.33	0.33	0.37	0.47	0.61	0.50	0.37	0.43
Recall-micro	0.89	0.89	0.89	0.89	0.87	0.91	0.89	0.89
Recall-weighted	0.89	0.89	0.89	0.89	0.87	0.91	0.89	0.89
F1-score-macro	0.32	0.31	0.38	0.50	0.57	0.57	0.38	0.43
F1-score-micro	0.89	0.89	0.89	0.89	0.87	0.91	0.89	0.89
F1-score-weighted	0.83	0.83	0.85	0.87	0.88	0.89	0.85	0.86
Average	0.72	0.70	0.76	0.78	0.79	0.83	0.77	

**Fig. 14:**
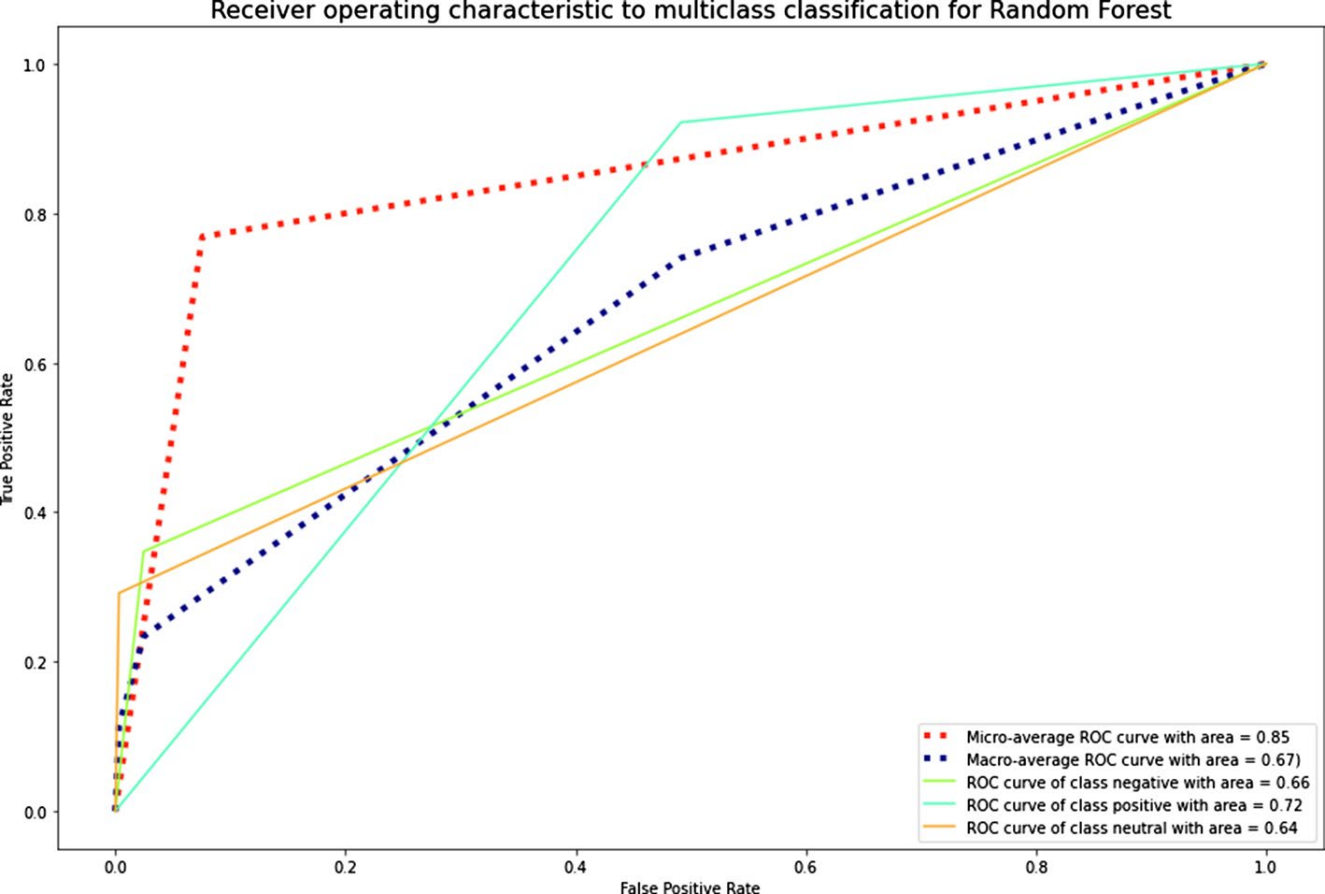
A ROC curve for Russian texts of an RF algorithm

**Fig. 15:**
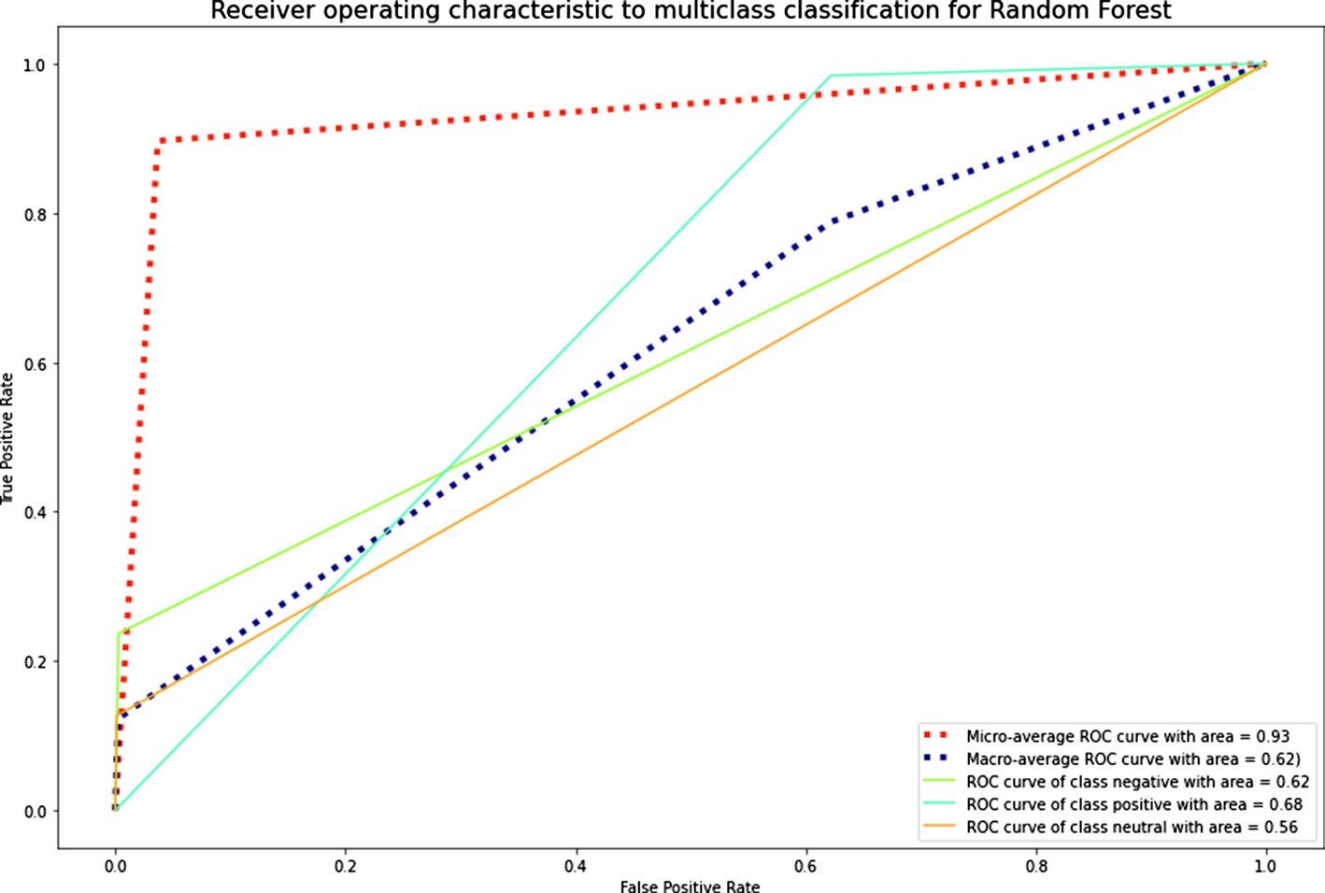
A ROC curve for Kazakh texts of an RF algorithm

The results showed that imbalanced classes had the lowest values of precision-macro, recall-macro, and F1-score-macro for SVM. NB, LR, k-NN, and XGBoost also demonstrated low results of the recall-macro and F1-score-macro metrics. RF, LR, and DT had the best average values for the imbalanced Russian texts. RF, DT, and k-NN were the best for the imbalanced Kazakh texts. Generally, RF was the best among all ML algorithms for both datasets. The graphics of AUC–ROC curves for an RF algorithm for the Russian and Kazakh texts are shown in Figs. 14 and 15.

The results of the classification of the oversampled Russian and Kazakh datasets are shown in Tables 5 and 6.

**Table 5:** The classification metrics for the oversampled Russian texts

Classifier	NB	SVM	LR	k-NN	DT	RF	XGBoost	Average
Accuracy	0.71	0.60	0.84	0.67	0.91	0.95	0.64	0.76
Precision-macro	0.73	0.61	0.84	0.77	0.91	0.95	0.64	0.78
Precision-micro	0.71	0.60	0.84	0.67	0.91	0.95	0.64	0.76
Precision-weighted	0.73	0.61	0.84	0.77	0.91	0.95	0.64	0.78
Recall-macro	0.71	0.60	0.84	0.66	0.91	0.95	0.64	0.76
Recall-micro	0.71	0.60	0.84	0.67	0.91	0.95	0.64	0.76
Recall-weighted	0.71	0.60	0.84	0.67	0.91	0.95	0.64	0.76
F1-score-macro	0.71	0.59	0.84	0.65	0.90	0.95	0.63	0.75
F1-score-micro	0.71	0.60	0.84	0.67	0.91	0.95	0.64	0.76
F1-score-weighted	0.71	0.59	0.84	0.65	0.90	0.95	0.63	0.75
Average	0.71	0.60	0.84	0.69	0.91	0.95	0.64

**Table 6:** The classification metrics for the oversampled Kazakh texts

Classifier	NB	SVM	LR	k-NN	DT	RF	XGBoost	Average
Accuracy	0.84	0.59	0.93	0.93	0.96	0.99	0.73	0.85
Precision-macro	0.85	0.59	0.93	0.94	0.96	0.99	0.73	0.86
Precision-micro	0.84	0.59	0.93	0.93	0.96	0.99	0.73	0.85
Precision-weighted	0.84	0.59	0.93	0.94	0.96	0.99	0.73	0.85
Recall-macro	0.84	0.59	0.93	0.93	0.96	0.99	0.73	0.85
Recall-micro	0.84	0.59	0.93	0.93	0.96	0.99	0.73	0.85
Recall-weighted	0.84	0.59	0.93	0.93	0.96	0.99	0.73	0.85
F1-score-macro	0.84	0.55	0.93	0.93	0.96	0.99	0.73	0.85
F1-score-micro	0.84	0.59	0.93	0.93	0.96	0.99	0.73	0.85
F1-score-weighted	0.84	0.54	0.93	0.93	0.96	0.99	0.73	0.85
Average	0.84	0.58	0.93	0.93	0.96	0.99	0.73	

**Fig. 16:**
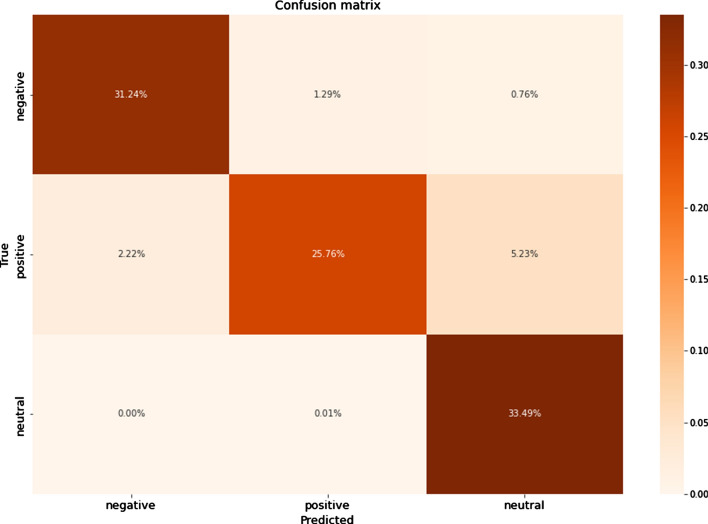
A confusion matrix for Russian texts of a DT algorithm

**Fig. 17:**
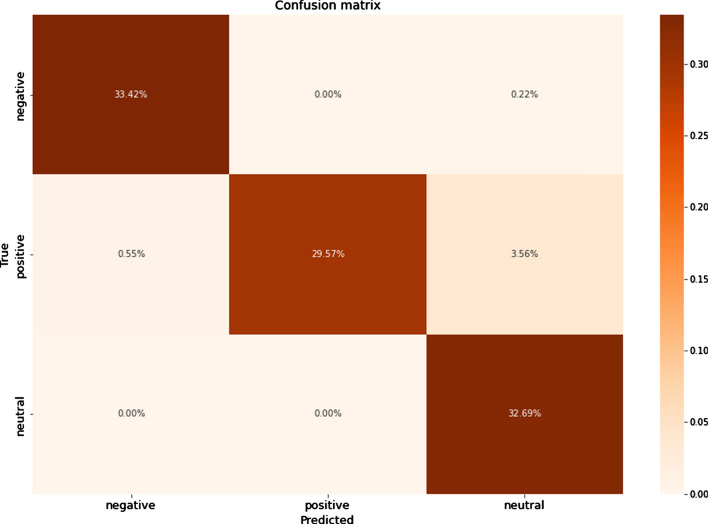
A confusion matrix for Kazakh texts of a DT algorithm

The results showed that the oversampling technique significantly improved the metrics values for all ML algorithms. Among them, DT and RF were essentially superior to others in the Russian texts. DT, RF, k-NN, and LR were all good for classifying the Kazakh texts. The graphics of confusion matrices for a DT algorithm for the Russian and Kazakh texts are shown in Figs. 16 and 17.

The results of the classification of the SMOTE Russian and Kazakh datasets are shown in Tables 7 and 8.

**Table 7:** The classification metrics for the SMOTE Russian texts

Classifier	NB	SVM	LR	k-NN	DT	RF	XGBoost	Average
Accuracy	0.67	0.64	0.85	0.69	0.83	0.91	0.69	0.75
Precision-macro	0.71	0.63	0.85	0.79	0.84	0.91	0.68	0.77
Precision-micro	0.67	0.64	0.85	0.69	0.83	0.91	0.69	0.75
Precision-weighted	0.71	0.63	0.85	0.79	0.84	0.91	0.68	0.77
Recall-macro	0.67	0.64	0.85	0.69	0.83	0.91	0.69	0.75
Recall-micro	0.67	0.64	0.85	0.69	0.83	0.91	0.69	0.75
Recall-weighted	0.67	0.64	0.85	0.69	0.83	0.91	0.69	0.75
F1-score-macro	0.67	0.63	0.85	0.65	0.82	0.91	0.68	0.74
F1-score-micro	0.67	0.64	0.85	0.69	0.83	0.91	0.69	0.75
F1-score-weighted	0.67	0.63	0.85	0.65	0.82	0.91	0.68	0.74
Average	0.68	0.64	0.85	0.70	0.83	0.91	0.69

**Table 8:** The classification metrics for the SMOTE Kazakh texts

Classifier	NB	SVM	LR	k-NN	DT	RF	XGBoost	Average
Accuracy	0.85	0.58	0.93	0.78	0.92	0.98	0.72	0.82
Precision-macro	0.85	0.58	0.93	0.84	0.92	0.98	0.72	0.83
Precision-micro	0.85	0.58	0.93	0.78	0.92	0.98	0.72	0.82
Precision-weighted	0.85	0.58	0.93	0.84	0.92	0.98	0.72	0.83
Recall-macro	0.85	0.58	0.93	0.79	0.92	0.98	0.72	0.82
Recall-micro	0.85	0.58	0.93	0.78	0.92	0.98	0.72	0.82
Recall-weighted	0.85	0.58	0.93	0.78	0.92	0.98	0.72	0.82
F1-score-macro	0.85	0.54	0.93	0.75	0.92	0.98	0.71	0.81
F1-score-micro	0.85	0.58	0.93	0.78	0.92	0.98	0.72	0.82
F1-score-weighted	0.85	0.54	0.93	0.75	0.92	0.98	0.71	0.81
Average	0.85	0.57	0.93	0.79	0.92	0.98	0.72	

**Fig. 18:**
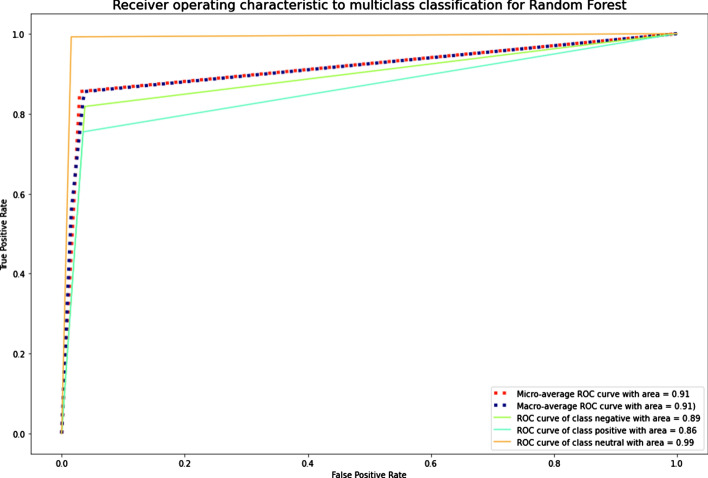
A ROC curve for Russian texts of an RF algorithm

**Fig. 19:**
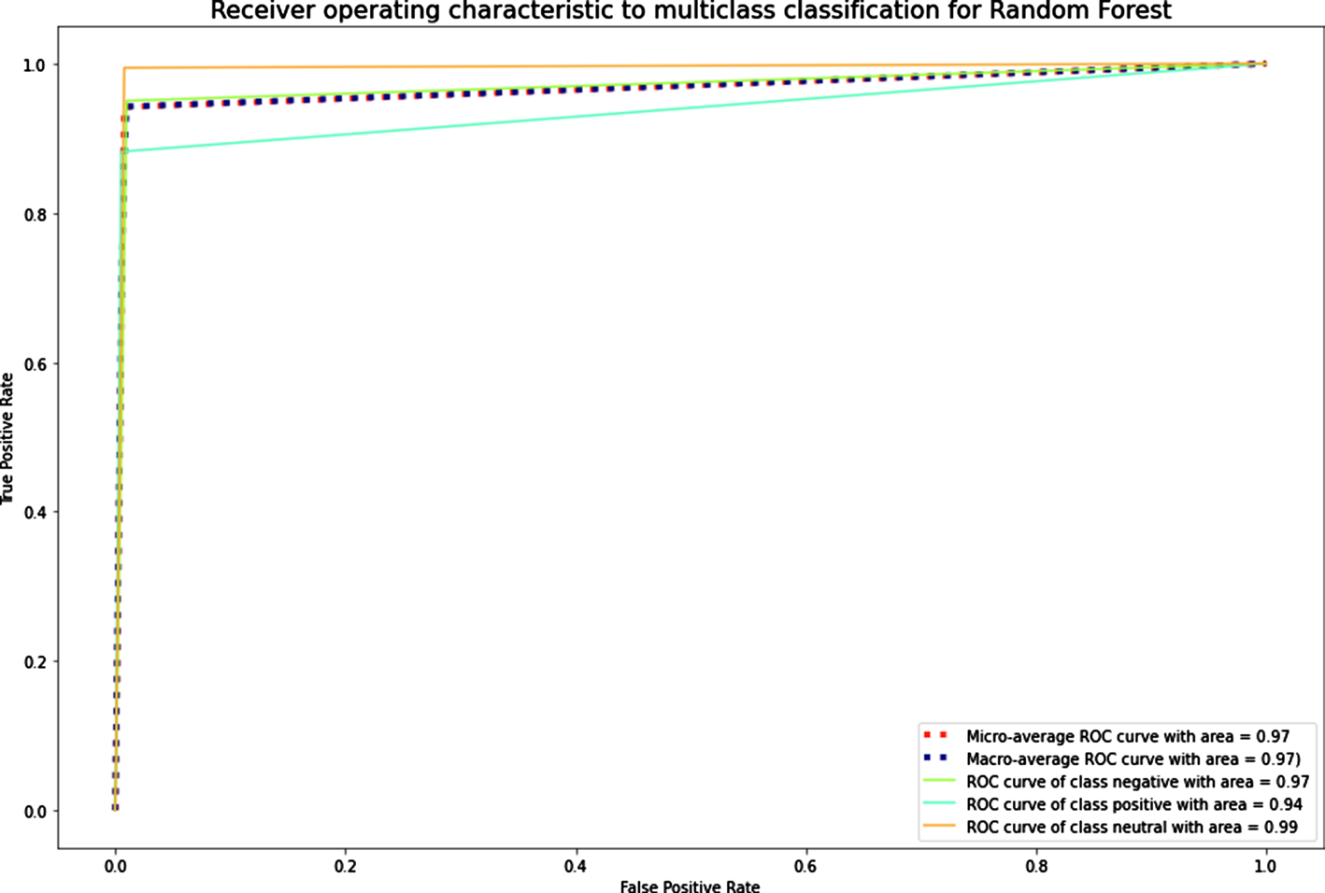
A ROC curve for Kazakh texts of an RF algorithm

The results demonstrated that the SMOTE technique also improved the metrics values as the Random oversampling technique. DT and RF outperformed other ML algorithms in classifying the datasets. The graphics of AUC–ROC curves for an RF algorithm for the Russian and Kazakh texts are shown in Figs. 18 and 19.

The results of the classification of the undersampled Russian and Kazakh datasets are shown in Tables 9 and 10.

**Table 9:** The classification metrics for the undersampled Russian texts

Classifier	NB	SVM	LR	k-NN	DT	RF	XGBoost	Average
Accuracy	0.57	0.53	0.72	0.39	0.63	0.71	0.62	0.60
Precision-macro	0.66	0.55	0.72	0.60	0.65	0.72	0.62	0.65
Precision-micro	0.57	0.53	0.72	0.39	0.63	0.71	0.62	0.60
Precision-weighted	0.66	0.55	0.72	0.61	0.65	0.72	0.63	0.65
Recall-macro	0.57	0.54	0.72	0.40	0.63	0.72	0.63	0.60
Recall-micro	0.57	0.53	0.72	0.39	0.63	0.71	0.62	0.60
Recall-weighted	0.57	0.53	0.72	0.39	0.63	0.71	0.62	0.60
F1-score-macro	0.56	0.44	0.71	0.30	0.62	0.71	0.62	0.57
F1-score-micro	0.57	0.53	0.72	0.39	0.63	0.71	0.62	0.60
F1-score-weighted	0.56	0.44	0.71	0.30	0.62	0.71	0.61	0.56
Average	0.59	0.52	0.72	0.42	0.63	0.71	0.62

**Table 10:** The classification metrics for the undersampled Kazakh texts

Classifier	NB	SVM	LR	k-NN	DT	RF	XGBoost	Average
Accuracy	0.53	0.58	0.72	0.63	0.70	0.74	0.67	0.65
Precision-macro	0.66	0.61	0.72	0.63	0.72	0.76	0.67	0.68
Precision-micro	0.53	0.58	0.72	0.63	0.70	0.74	0.67	0.65
Precision-weighted	0.67	0.61	0.72	0.63	0.71	0.76	0.67	0.68
Recall-macro	0.54	0.58	0.72	0.63	0.70	0.74	0.67	0.65
Recall-micro	0.53	0.58	0.72	0.63	0.70	0.74	0.67	0.65
Recall-weighted	0.53	0.58	0.72	0.63	0.70	0.74	0.67	0.65
F1-score-macro	0.51	0.54	0.71	0.62	0.69	0.73	0.67	0.64
F1-score-micro	0.53	0.58	0.72	0.63	0.70	0.74	0.67	0.65
F1-score-weighted	0.51	0.54	0.71	0.62	0.69	0.73	0.67	0.64
Average	0.55	0.58	0.72	0.63	0.70	0.74	0.67	

**Fig. 20:**
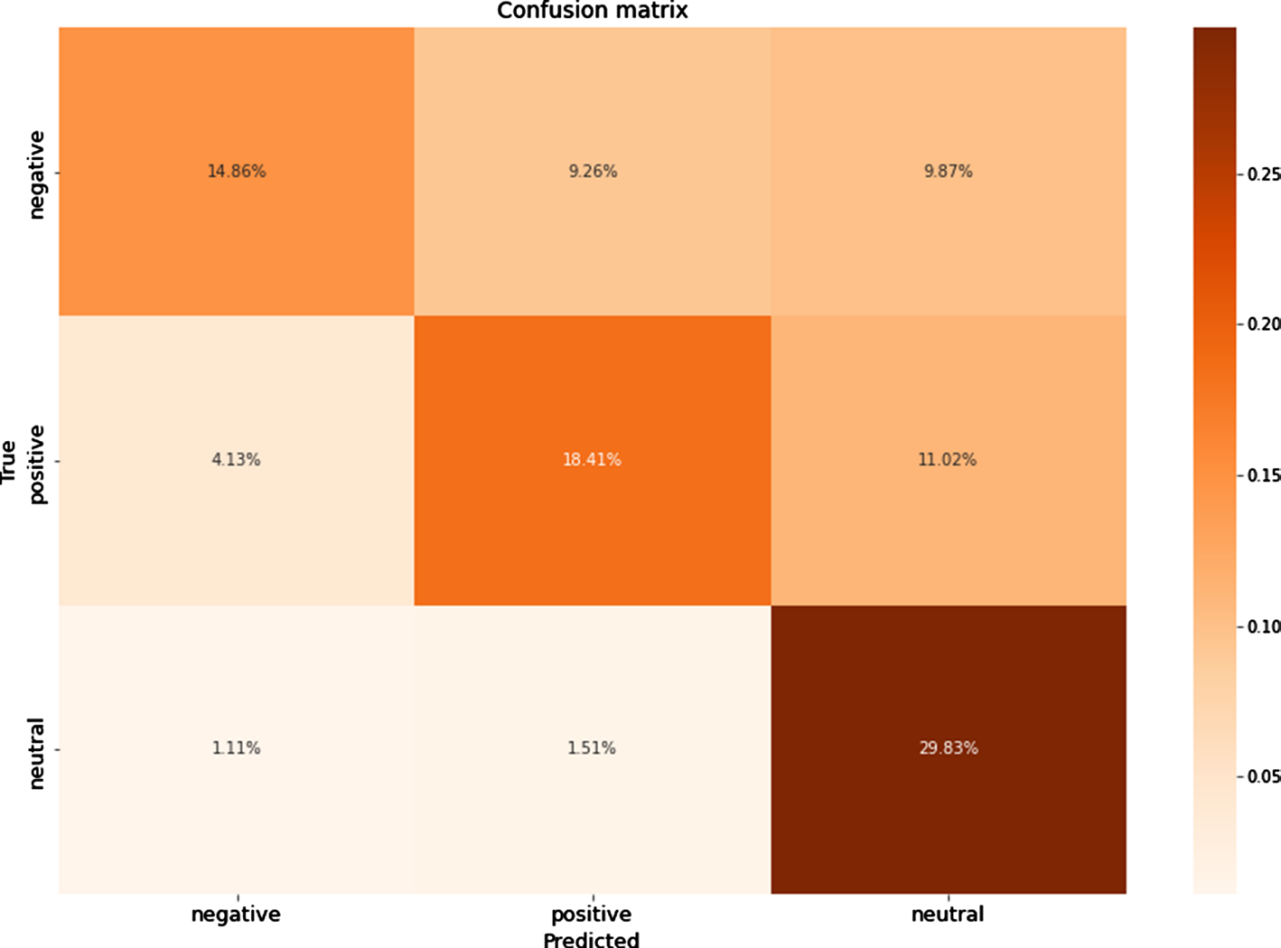
A confusion matrix for Russian texts of a DT algorithm

**Fig. 21:**
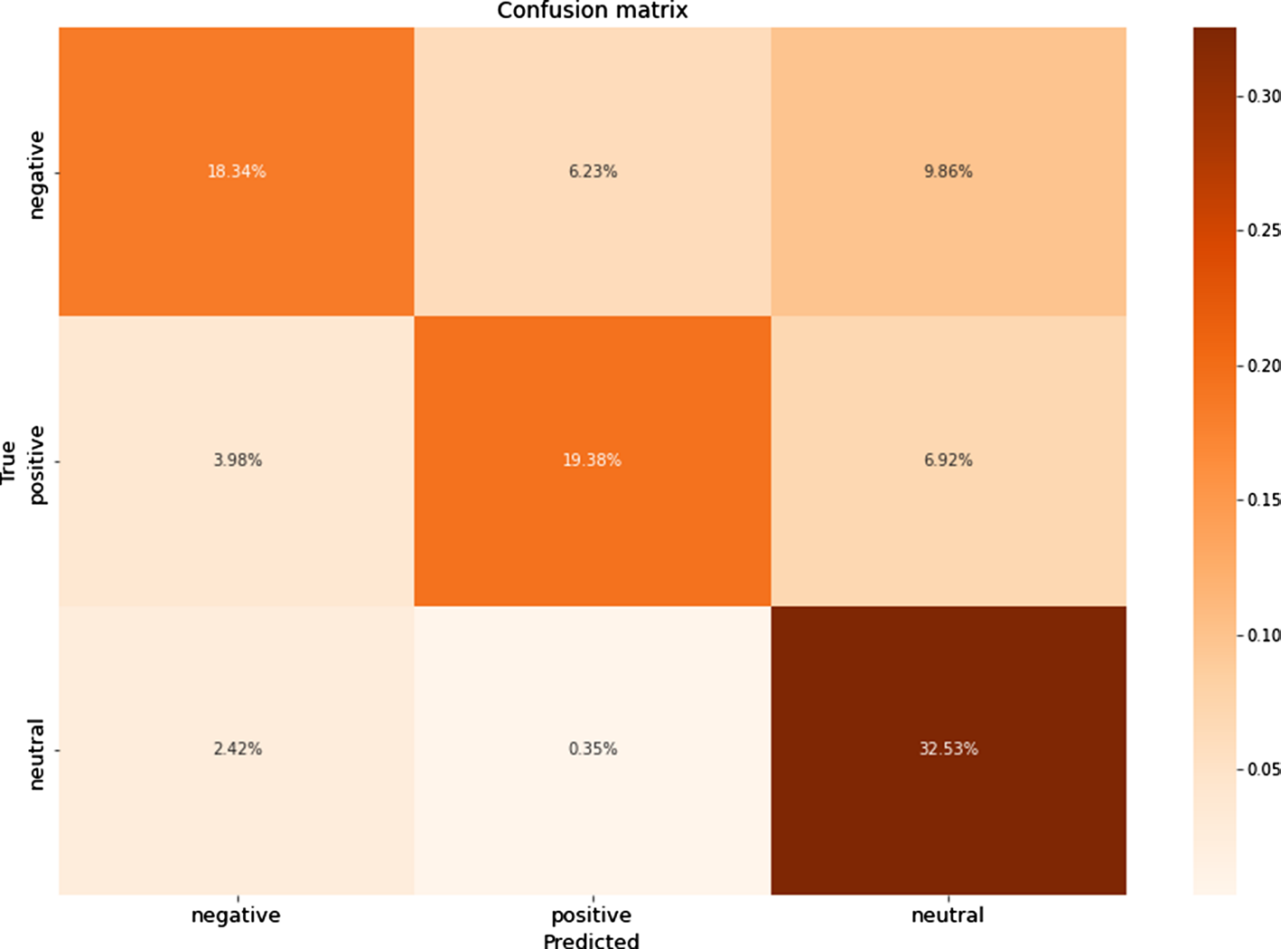
A confusion matrix for Kazakh texts of a DT algorithm

In the results, it could be seen that the values of the undersampled datasets dropped compared with the oversampled and SMOTE datasets. It is caused by the significant decrease in the sizes of the positive and negative classes to make them equal to the negative class. As in the previous experiments, the DT and RF classifiers showed the best results. The graphics of confusion matrices for a DT algorithm for the Russian and Kazakh texts are shown in Figs. 20 and 21.

**Fig. 22:**
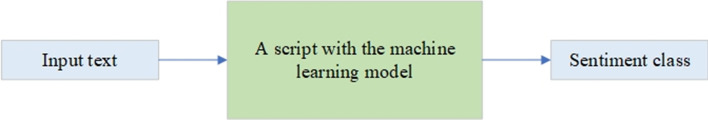
New text labeling

**Fig. 23:**
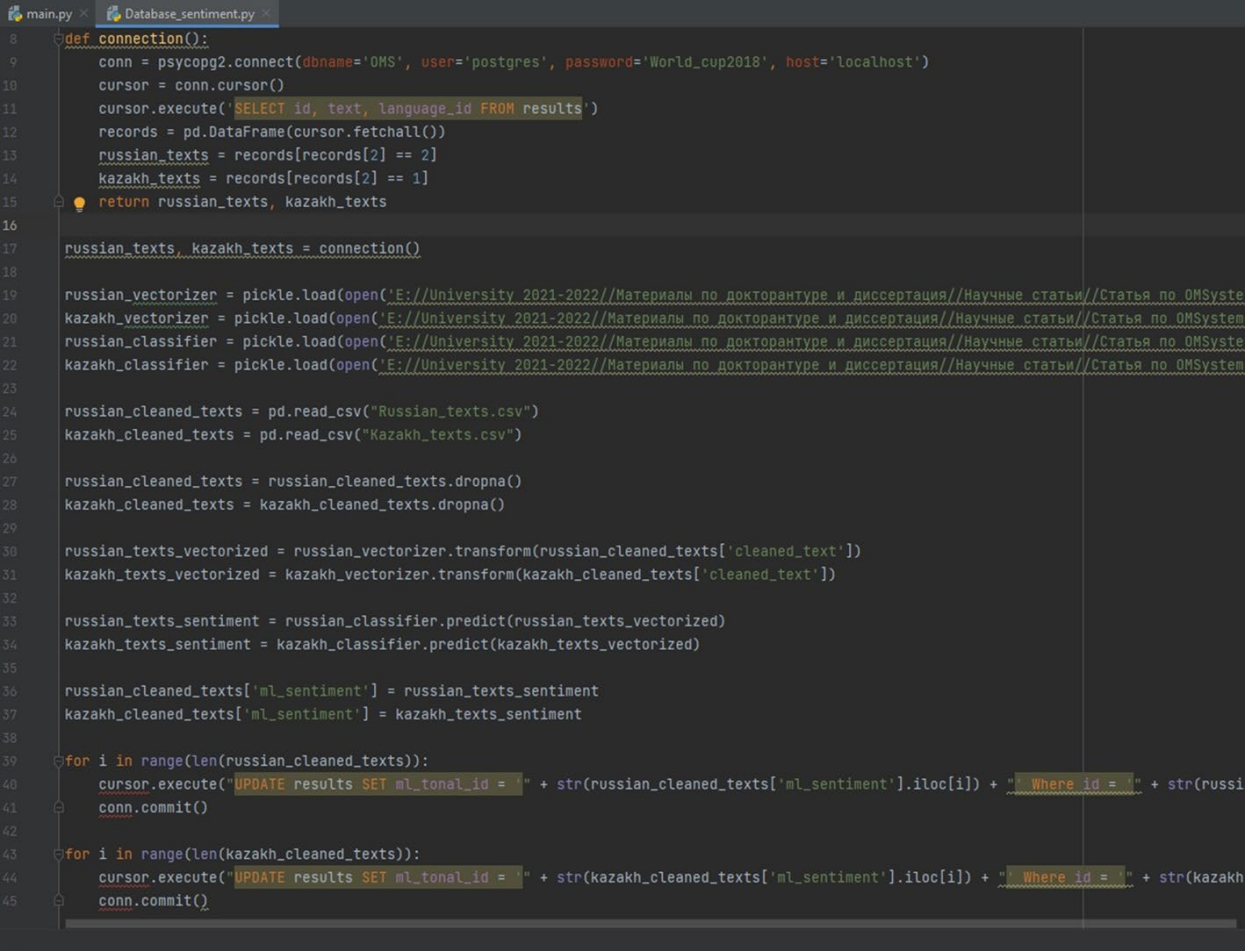
A text labeling script

All the built classification models showed that models trained on the imbalanced datasets achieved the lowest performance. The Random undersampling method gave average values of metrics. The reason for this is that the resulting models cannot fully use the entire dataset being significantly decreased in size. The Random oversampling and SMOTE models expectedly demonstrated the best results. Among ML algorithms, LR and DT reached the best performance. As RF uses multiple independent DTs, it is clear that it outperformed a single DT. Classification results for the Russian and Kazakh datasets are comparatively equal, with slightly better performance for the latter on the oversampled and SMOTE datasets having a smaller test size. When the RF and DT ML models are trained on the oversampled and SMOTE datasets, they are saved in the files using the Python pickle library. Then a script file that processes a new parsed text with the saved classification model is implemented. In this script, a new text is input data; the saved ML model is a data processing tool; a defined sentiment class of the text is output data. The output data is saved in the corresponding table of the PostgreSQL database of the OMSystem.If it is required to change the trained model, simple corrections to the script are to be made. When the database has grown significantly, the classification models need to be retrained, and the models are saved again.The scheme of labeling texts and the script are shown in Figs. 22 and 23.

The experimental results have also been compared to the social media SA papers. The comparison is shown in Table 11.

**Table 11:** The results comparison

Study	Specifications	Results
Our research	The first experiment explored the development of ML models. Totally, 132,523 texts on various topics, including Covid-19, were gathered	The best results of accuracy were achieved by DT (0.91–0.95) and RF (0.96–0.99) with the Random oversampling technique
Akpatsa et al. [16]	This paper analyzed topics, discussions, and concerns about Covid-19 vaccination using Twitter datasets. The final dataset contains 15,239 unique tweets	It achieved the following accuracies with an LR (0.83), an RF (0.83), an SVM (0.84), and an NB (0.77)
Yeasmin et al. [71]	This research explored Twitter datasets to analyze sentiments on the Covid-19 topic. The dataset included tweets from different states of the USA for 15 days. A total number of 832,528 tweets were gathered	The following results of classification were achieved with ML algorithms: an LR (0.91), an SVM (0.94), an NB (0.91), k-NN (0.90), a DT (0.96), a RF (0.97), and XGBoost (0.83)
Daradkeh et al. [72]	This paper describes SA of topics related to Covid-19 vaccine misinformation. A corpus of 40,359 tweets has been collected for the dates between January 2021 and March 2021	It got the following values of accuracy: a DT (0.81), an SVM (0.78), a k-NN (0.76), and an NB (0.74)
Mishra et al. [73]	This research paper analyzed the public’s sentiments towards the Covid-19 vaccination in India. The dataset included 5977 tweets before the second wave and 42,936 tweets after the second wave	The following values of accuracy were achieved: an LR (0.61), a DT (0.45), a k-NN (0.58), an RF (0.59), and an XGBoost (0.54)
Iwendi et al. [74]	This paper focuses on gathering real and fake news data on the topics related to Covid-19. The dataset consisted of 586 true news and 578 fake news and 1100 news articles and social media posts regarding Covid-19	The ML algorithms achieved the following values of accuracy: a k-NN (0.69), a DT (0.77), and AdaBoost (0.83)

### Defining the social mood on the topic of vaccination against Covid-19

A relevant topic of vaccination against coronavirus infection [75] is taken for analysis in the experimental part. This topic is very important due to the active vaccination [76] of people in the world and Kazakhstan. A large number of news articles have been written on this topic, and users actively comment on various issues related to it. The opinions of users stand out with positive, neutral, and negative sentiments. The experimental part chooses a list of keywords and phrases in the Russian language to monitor the corresponding topics. In the following description of the experiment, all words and phrases originally in the Russian language are translated into the English language for convenience and the right understanding. These keywords and phrases are “Vaccination in Kazakhstan,” Covid [76], Coronavirus [77, 78], Sputnik, “Russian vaccine,” Pfizer [79], QazVac, Hayat, Sinovac, Sinopharm [80], “Vaccine rejection,” “Fear of vaccination,” “Choice of the vaccine,” “Vaccine effectiveness,” “Lack of confidence in the vaccine,” and Tsoi (the last name of the Minister of Health of the Republic of Kazakhstan).

In the preprocessing step, all words are transformed to the lowercase register. Then punctuation marks, digits, and other special symbols that do not carry any significant meaning are removed. Additionally, it is required to delete frequent words (i.e., stop words such as ‘and,’ ‘or,’ ‘in,’ ‘on,’ ‘at,’ ‘for,’ etc.), which do not bring any significant meaning [50]. However, ‘to be’ and ‘is’ stop words are left because they are met in expressions such as “to be vaccinated,” “is vaccinated,” and others, which are important for the analyzed topic.

The stemming step reduces the number of words with similar meanings by eliminating affixes and endings to gain their roots. Russian words are processed by ‘SnowballStemmer’ from the Python NLTK library. The text vectorization step transforms texts into a numeric vector representation to which ML algorithms are applied [50]. The vectorization is done with the use of the TF-IDF metric that considers the importance of words in the text. After the texts are vectorized, the trained ML models are applied to label them in three sentiment classes.

Next, the number of words in texts and comments is counted, and the most frequently used ones are displayed in pivot tables. The OMSystem [50, 51] performs calculations for two periods: the 10th of January, 2021 to the 30th of May, 2021 (Table 12) and the 1st of July, 2021 to the 12th of August, 2021 (Table 14), and two groups of cities: Almaty (the largest city of Kazakhstan) and Nur-Sultan (the capital of Kazakhstan), and large regional cities of Kazakhstan. The choice of these cities for analysis was made due to several facts. First, the population of Almaty, Nur-Sultan, and other large cities is almost 100% covered with information technologies. Citizens of these cities are also the most active users of social networks, and their opinions are very important, reflecting the general trend in the country. It is also important to get the public’s opinion from different regional cities because the epidemiological situation with vaccination and the availability of vaccines significantly varied in all the regions of Kazakhstan. The stated dates of monitoring were chosen because the start of vaccination campaign of the vaccination against Covid-19 started in January 2021. The first phase of vaccination finished by the beginning of summer. In the first phase, only two vaccines called “Sputnik V” and QazVac were available. Then in May and June 2021, three more vaccines, Hayat-Vax, Sinovac, and Sinopharm, were imported. Nevertheless, in the second phase of vaccination, these vaccines quickly ran out in Almaty, Nur-Sultan, and some other cities. It resulted in a large number of negative user comments. So it was essential to monitor these two periods of the vaccination campaign to estimate the level of interest and social mood in this topic.

**Table 12:** Analysis by topics for period 1

Resource set	News portals, Vkontakte, Facebook, Instagram, Youtube			Vkontakte, Facebook, Instagram, Youtube			
Search period:	From “01/10/2021” to “05/30/2021”						
Location:	Cities of Almaty and Nur-Sultan			Large regional cities of Kazakhstan			
Number of results (texts + comments)	~ 19,340			~ 1228			
Number of texts	~ 4919			~ 122			
Number of comments	~ 14,421			~ 1106			
The level of social mood by results	**Positive**	**8944**		Positive	396		
	Negative	8152		**Negative**	**683**		
	Neutral	1082		Neutral	66		
	Undefined	1162		Undefined	83		
The level of social mood by texts	**Positive**	**3829**		**Positive**	**56**		
	Negative	960		Negative	43		
	Neutral	123		Neutral	11		
	Undefined	7		Undefined	12		
The level of social mood by comments	Positive	5115		Positive	340		
	**Negative**	**7192**		**Negative**	**640**		
	Neutral	959		Neutral	55		
	Undefined	1155		Undefined	71		
The level of topic discussion activity in society	~ 0.48%			~ 0.08%			
The level of interest in the topic in society	~ 491%			~ 12.2%			
**Engagement level**				**Engagement level**			
Views	~ 9M			~ 341K			
Comments	~ 14K			~ 1K			
Reposts	~ 2K			~ 249			
Likes	~ 32K			~ 2K			
Dislikes	~ 2K			~ 305			
Total engagement level	~ 9M			~ 345K			
**Popular words**				**Popular words**			
**By texts**		**By comments**		**By texts**		**By comments**	
**Word**	**Frequency of use**	**Word**	**Frequency of use**	**Word**	**Frequency of use**	**Word**	**Frequency of use**
Coronavirus	2374 (3.51%)	To be	1598 (1.38%)	Coronavirus	118 (1.29%)	To be	148 (1.52%)
Kazakhstan	1811 (2.68%)	**Vaccine**	**1112 (0.96%)**	To be	113 (1.24%)	Person	138 (1.42%)
Vaccine	824 (1.22%)	Person	1097 (0.94%)	Area	112 (1.23%)	**Vaccine**	**105 (1.08%)**
Person	653 (0.96%)	Can	564 (0.48%)	Kazakhstan	101 (1.11%)	People	92 (0.94%)
Covid-19	540 (0.80%)	Is	532 (0.46%)	Vaccine	68 (0.74%)	Kazakhstan	62 (0.63%)
Day	526 (0.77%)	Kazakhstan	477 (0.41%)	Aktyubinsk	59 (0.64%)	Year	53 (0.54%)
Vaccination	524 (0.77%)	**Necessary**	**472 (0.40%)**	Year	55 (0.60%)	**Necessary**	**44 (0.45%)**
News	502 (0.74%)	Year	432 (0.37%)	Person	53 (0.58%)	Virus	42 (0.43%)
Almaty	443 (0.65%)	People	415 (0.35%)	Vaccination	52 (0.57%)	Country	41 (0.42%)
New	433 (0.64%)	Virus	370 (0.32%)	Tenge	49 (0.53%)	Can	40 (0.41%)
Country	427 (0.63%)	To speak	337 (0.29%)	Reference	48 (0.52%)	**Vaccination**	**40 (0.41%)**
To be	394 (0.58%)	Country	327 (0.28%)	Zone	46 (0.50%)	Power	35 (0.36%)
Case	371 (0.54%)	**To do**	**327 (0.28%)**	Case	42 (0.46%)	Good	29 (0.29%)
The first	358 (0.53%)	**Vaccination**	**325 (0.28%)**	To attach	40 (0.43%)	Russia	29 (0.29%)
Area	325 (0.48%)	To tell	317 (0.27%)	Later	39 (0.42%)	Simply	29 (0.29%)
Zone	310 (0.45%)	To want	312 (0.27%)	Child	36 (0.39%)	Covid	29 (0.29%)
Ministry of Health	282 (0.41%)	Nobility	297 (0.25%)	Can	34 (0.37%)	Child	28 (0.28%)
To reveal	281 (0.41%)	Money	286 (0.24%)	Doctor	33 (0.36%)	Inoculation	28 (0.28%)
Tsoi	274 (0.40%)	Another	281 (0.24%)	Healthcare	32 (0.35%)	World	27 (0.27%)
Year	271 (0.40%)	Good	279 (0.24%)	Region	31 (0.34%)	Quarantine	27 (0.27%)

The bold text indicates the highest sentiment of results, texts, and comments, and the most important words on the topic of vaccination against the coronavirus disease

**Fig. 24:**
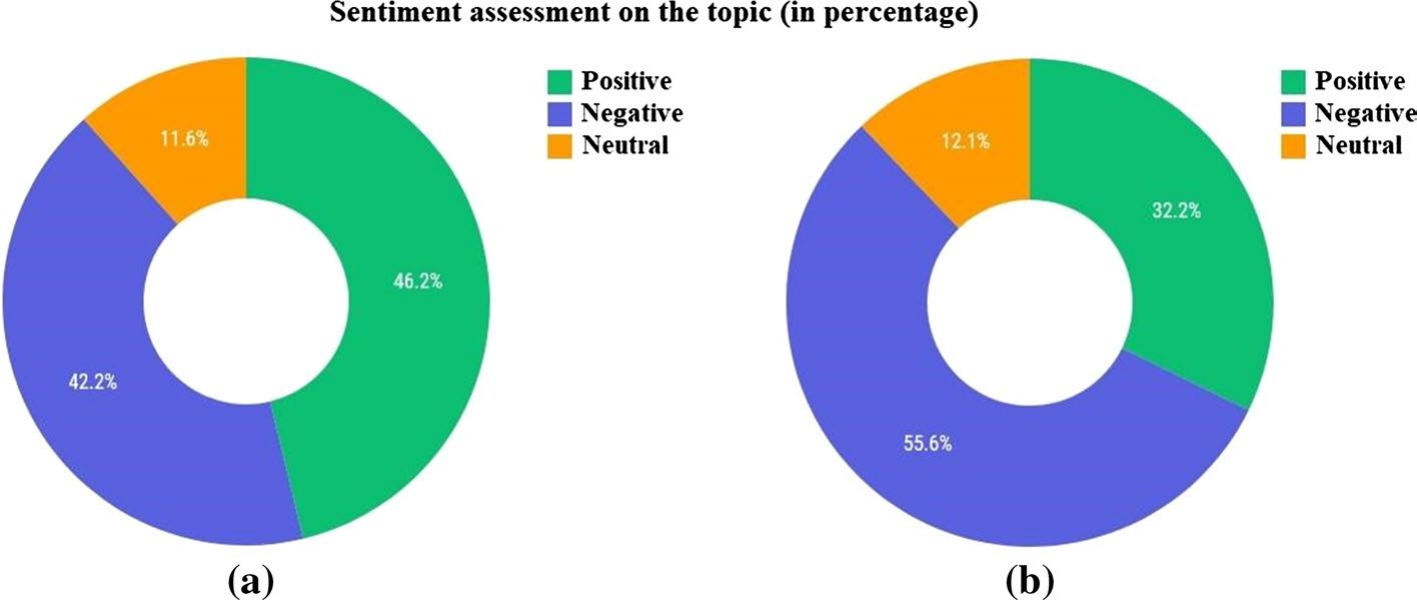
Evaluation of the sentiment of the first period—**a** Almaty and Nur-Sultan, **b** large regional cities

The sentiment charts of the first period for the cities of Almaty and Nur-Sultan and large regional cities are shown in Fig. 24.

Based on the results of the analysis of Table 12, it is possible to evaluate the content of texts and comments, taking into account the list of the most popular words. Furthermore, looking at the analysis of popular words in the context of regional cities, we will see that they coincide with the content in the cities of Almaty and Nur-Sultan. After the most popular words on the topic are highlighted, the results are generally evaluated by the level of topic discussion activity, the level of interest in the topic, and the level of social mood. According to the obtained results, the level of interest in this topic is significantly higher in the cities of Almaty and Nur-Sultan (491%) than in other large regional cities (12.2%). In addition, the level of topic discussion is also higher in the two main cities of the country (0.48%) than in other ones (0.08%). The level of social mood of texts and comments differs significantly, with the positive sentiment prevailing over the negative sentiment in texts and the negative sentiment prevailing over the positive sentiment in comments. It shows that texts on social media positively cover the topic of vaccination, while people’s attitude is the opposite. After the system had created a summary analysis, the gained texts and comments were manually read and investigated. Their examples are presented in Table 13. The public reacted negatively to all governmental measures related to the vaccination campaign in the winter and spring seasons, showing their distrust of the newly adopted policies and calling for the rejection of vaccination.

**Table 13:** Texts and comments for period 1

No.	Date	Sentiment	Text	Sentiment	Comments
1	19-01-2021	Positive	The Head of the Government instructed the Ministry of Health of the Republic of Kazakhstan to ensure the readiness of medical organizations for the start of the mass vaccination of the population with the “Sputnik V” vaccine from February 1	Negative	We do not need your vaccine; go to poison others with these chemicals
				Negative	Look for idiots elsewhere. Madhouse
				Negative	Experiments on humans are like this, especially when all sane scientists deny the vaccine’s effectiveness. So it is time to be vaccinated!
				Negative	Madness! Even in Russia, they did not really test it. Did they decide to test it on the Kazakhs? What a madhouse?
				Positive	Ready to become a test subject for a fee. Where to go?
2	30-01-2021	Positive	First of all, vaccines against COVID-19 will be sent to health workers in eight regions of Kazakhstan, in which there is a high incidence of coronavirus. On Monday, February 1, vaccination against coronavirus starts, but vaccines have not yet been brought to the Aktobe region	Negative	Now we will look before the vaccine and after
				Positive	Soon, vaccinations will start everywhere, and it will be much more difficult for the coronavirus to spread. So the epidemic will end
				Negative	Why do not we start with the deputies?
				Positive	The entire Government with their families must be at the forefront. May they get the best, we undoubtably agree this time
3	08-05-2021	Positive	In Kazakhstan, 34% of residents have changed their attitude towards vaccination against COVID-19 for the better. At the same time, 23% are skeptical against vaccinations. 9% have recently changed their minds in a negative direction. 4% do not trust the vaccine, 3% do not dare to get vaccinated, as they recently got sick. Who do you belong to?	Negative	The data is not exact. More than half of the population of Kazakhstan do not believe
				Negative	Where do these statistics come from? For example, no one asked me)
				Negative	I did it because I wanted to save my family. The opinions of others do not matter, but my health and safety do
				Neutral	There is no way
				Negative	Suicidal people are getting vaccinated
4	01-02-2021	Positive	Kazakhstanis are intended to be classified by color in terms of whether they passed the polymerase chain reaction (PCR) test and what the result was, zakon.kz reports. According to the press service of the Republic of Kazakhstan, the data will be reflected in the “Ashyq” application developed by the Ministry of Digital Development, Innovation and Aerospace Industry jointly with the Ministry of Health of the Republic of Kazakhstan	Negative	I am crazy with all sorts of this bullshit to torture the people
				Negative	Well! It is straight racism: yellow and red. I disagree
				Negative	It is a total control under the guise of coronavirus
				Negative	“Divide and conquer” is a working scheme from the ancient time
				Negative	Scumbugs! I knew it would come to this!
5	26-01-2021	Positive	Mass vaccination of the population against COVID-19 will begin in Kazakhstan on February 1, Kazakh Health Minister Alexei Tsoi said during a government meeting on Tuesday. It is planned to vaccinate up to six million people by the end of the year	Negative	Are we their guinea pigs or what? Go away. Check your vaccine to the end first, then to the people
				Negative	It is necessary to start with the ministers and deputies. Whoever survives will remain in office, who does not survive, and to hell with them!
				Negative	They want to test the effectiveness of the vaccine on us
				Negative	Let them first try this vaccine on themselves. We did not invent this infection; it was not for us to die for it
				Positive	Yes. Nevermind. As they said, it will be “finally,” and vaccination is already in full swing

**Fig. 25:**
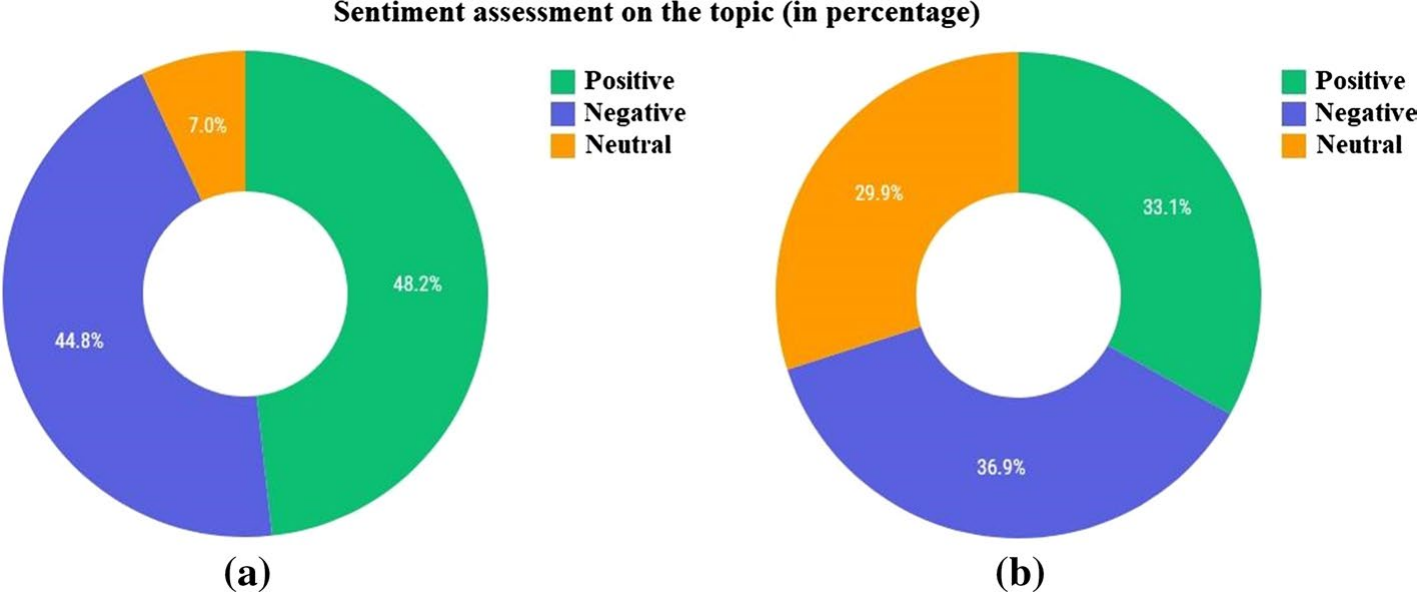
Evaluation of the sentiment of the second period—**a** Almaty and Nur-Sultan, **b** large regional cities

The sentiment charts of the second period for the cities of Almaty and Nur-Sultan and large regional cities are shown in Fig. 25.

The analysis of Table 14 suggests that there remains a high level of public interest in the topic during the summer. The level of interest in this topic is higher in the cities of Almaty and Nur-Sultan (128%) than in large regional cities (6.9%). The level of topic discussion activity is lower than in period 1. It is caused by fewer comments on the considered topics during a shorter time of monitoring. The following values are gained in the context of cities: 0.01% for Almaty and Nur-Sultan and 0.03% for the large regional cities. The level of the social mood of texts and comments shows a situation similar to period 1. This period’s obtained texts and comments were also manually analyzed to reveal interesting points. It is noted that texts cover the planned children’s vaccination topics, the appearance of new strains of coronavirus, the increase in the number of cases of unvaccinated people’s disease, and the supply of a new Chinese vaccine to the country. The corresponding examples of the texts and comments are presented in Table 15.

**Table 14:** Analysis by topics for period 2

Resource set	News portals, Vkontakte, Facebook, Instagram, Youtube			Vkontakte, Facebook, Instagram, Youtube			
Search period:	from “07/01/2021” to “08/12/2021”						
Location:	Cities of Almaty and Nur-Sultan			Large regional cities of Kazakhstan			
Number of results (texts + comments)	~ 2133			~ 157			
Number of texts	~ 1285			~ 69			
Number of comments	~ 848			~ 88			
The level of social mood by results	Positive	1029		Positive	52		
	Negative	955		Negative	58		
	Neutral	80		Neutral	10		
	Undefined	69		Undefined	37		
The level of social mood by texts	Positive	739		Positive	21		
	Negative	544		Negative	18		
	Neutral	1		Neutral	5		
	Undefined	1		Undefined	25		
The level of social mood by comments	Positive	290		Positive	31		
	Negative	411		Negative	40		
	Neutral	79		Neutral	5		
	Undefined	68		Undefined	12		
The level of topic discussion activity in society	~ 0.01%			~ 0.03%			
The level of interest in the topic in society	~ 128%			~ 6.9%			
**Engagement level**				**Engagement level**			
Views	~ 34K			~ 42K			
Comments	~ 848			~ 97			
Reposts	~ 825			~ 46			
Likes	~ 2K			~ 123			
Dislikes	~ 35			~ 0			
Total engagement level	~ 38K			~ 42K			
**Popular words**				**Popular words**			
**By texts**		**By comments**		**By texts**		**By comments**	
**Word**	**Frequency of consumption**	**Word**	**Frequency of consumption**	**Word**	**Frequency of consumption**	**Word**	**Frequency of consumption**
To be	1786 (1.00%)	**Vaccine**	**143 (1.59%)**	Coronavirus	52 (2.15%)	Person	11 (1.59%)
Kazakhstan	1630 (0.91%)	Person	82 (0.91%)	Reference	44 (1.82%)	**Vaccine**	**11 (1.59%)**
Person	1493 (0.83%)	To be	63 (0.70%)	To attach	40 (1.66%)	To be	11 (1.59%)
Year	1268 (0.71%)	**Vaccination**	**46 (0.51%)**	Area	36 (1.49%)	Simply	8 (1.16%)
Coronavirus	1213 (0.68%)	Kazakhstan	39 (0.43%)	Strain	22 (0.91%)	People	6 (0.87%)
**Vaccination**	**1201 (0.67%)**	Later	38 (0.42%)	Pavlodar	22 (0.91%)	Though	4 (0.58%)
**Vaccine**	**1018 (0.57%)**	**Child**	33 (0.36%)	Kazakhstan	21 (0.87%)	Level	4 (0.58%)
Case	808 (0.45%)	Can	33 (0.36%)	Heading	20 (0.83%)	To buy	4 (0.58%)
Country	750 (0.42%)	To speak	33 (0.36%)	**Vaccine**	**20 (0.83%)**	Proper	4 (0.58%)
Infection	724 (0.40%)	Necessary	30 (0.33%)	Url	20 (0.83%)	Virus	4 (0.58%)
Covid-19	722 (0.40%)	To know	30 (0.33%)	To be	19 (0.78%)	Guilty	4 (0.58%)
Can	692 (0.38%)	Is	30 (0.33%)	Year	17 (0.70%)	Strain	3 (0.43%)
Area	674 (0.37%)	Covid	29 (0.32%)	Person	16 (0.66%)	**To make**	**3 (0.43%)**
July	638 (0.35%)	People	27 (0.30%)	Can	14 (0.58%)	**Inoculation**	**3 (0.43%)**
More	635 (0.35%)	**To do**	**24 (0.26%)**	**Vaccination**	**14 (0.58%)**	In a row	3 (0.43%)
Day	633 (0.35%)	Year	24 (0.26%)	Health care	13 (0.53%)	Life	3 (0.43%)
Work	631 (0.35%)	Doctor	24 (0.26%)	Pavlodar	12 (0.49%)	Any	3 (0.43%)
New	630 (0.35%)	**To be ill**	**24 (0.26%)**	Doctor	12 (0.49%)	Small	3 (0.43%)
Coronavirus	612 (0.34%)	To tell	23 (0.25%)	To work	11 (0.45%)	To know	3 (0.43%)
Patient	603 (0.33%)	Virus	21 (0.23%)	To become	10 (0.41%)	Delta	3 (0.43%)

The bold text indicates the highest sentiment of results, texts, and comments, and the most important words on the topic of vaccination against the coronavirus disease

**Table 15:** Texts and comments for period 2

No.	Date	Sentiment	Text	Sentiment	Comments
1	21-07-2021	Positive	It is planned to start vaccination of children against coronavirus in Kazakhstan at the end of this year. What do they plan to vaccinate with, and will vaccination be voluntary?	Negative	People stand up to protect children. Healthcare is not able to protect children from vaccine refusal
				Positive	In the USA, all children are vaccinated. If we can protect our children, why not? One of my acquaintances received the 2nd dose. She is a 14-year-old girl. Everything is fine. Everybody there voluntarily vaccinates children. We always lag behind. They tell us to take a step ahead, but we take two steps back. Sadly. Therefore, we do not grow, and we do not develop
				Negative	You must vaccinate yours!!! If you do not have brains, your children do not have one either!
2	26-07-2021	Positive	We have 84% of our intensive care beds filled. They are loaded with patients who have not received vaccination against coronavirus infection and are now in severe condition—248 patients. Of these, 77 people are in extremely serious condition. This number scares us as doctors. We are reaching the peak that was last summer,” said the head of the public health department of the capital, Timur Muratov	Negative	It is for those anti-vaccinators who can read and hear not only their cries about freedom. As soon as each of them understands the inhuman basis of personal freedom, as opposed to the freedom of others, or rather other people, he/she is obliged to think
				Negative	The relatives of the deceased can sue the Shymkent anti-vaccinator (I forgot her name, sorry), which actively urges everyone to refuse vaccination
				Negative	Let us gather money for the monuments to the killer doctors! Who sold out for premiums and killed people with the vaccine!!! They also lie!!! I am waiting for the heavenly punishment for you !!!
3	10-08-2021	Positive	The first lot of the Chinese vaccine Sinopharm arrived in Kazakhstan on August 10, 2021. Following the negotiations with the People's Republic of China, an aircraft with the first batch of Sinopharm vaccine against coronavirus arrived in Almaty at the warehouses of the SK-Pharmacy Single Distributor	Positive	Good vaccine! It was recognized by WHO and Europe. Vaccinate. Health to all
				Positive	Hayat is a good vaccine, so this one too. It is judging by my own example
				Negative	I doubt very much that WHO is responsible for our health and life
				Negative	Perhaps the quality of this vaccine is good (I do not argue). Just answer what it is made of, what is included in the composition?
				Positive	Hooray, I'm going to put it. Do not miss the vaccine that came at the expense of the people
4	11-08-2021	Negative	The Ministry of Health of the Republic of Kazakhstan notes that 99.9% of the incidence of Covid-19 falls on unvaccinated citizens. In assessing the effectiveness of vaccination, it was found that 99.9% of the incidence of coronavirus infection falls on unvaccinated, while the proportion of patients after vaccination was only 0.1%, such data reported today by the Minister of Health Alexei Tsoi at a meeting of the Government	Negative	And it is true! Three friends are now in the hospital. There are no vaccinated people in the wards
				Negative	What is the percentage of re-illnesses? If such statistics do not even exist, then this means that there are no more patients, and then the question arises, why vaccinate those who have already been ill?
5	02-07-2021	Negative	The “Indian” strain was found in all regions of Kazakhstan and the cities of Nur-Sultan, Almaty, Shymkent, zakon.kz reports. According to the Ministry of Health, the department carried out PCR screening of positive laboratory samples obtained from patients with coronavirus infection (CVI)	Negative	There is no Indian strain. They said officially. It is ours who are lying to make people run to shoot up drugs. The day before yesterday, it was in 4 regions, and yesterday it was in all. Walked in the wind
				Positive	Well, there is no point in getting vaccinated!
				Negative	Do not write Indian. People in India know how upset it is
				Negative	The Hindus themselves say there is no such thing
				Positive	These viruses appear abroad, but they come to us to die

The experimental results have been extensively studied and analyzed to understand the root of the public’s negative sentiment. Based on the data obtained by the OMSystem, it was concluded that Kazakhstanis, for the most part, do not trust the governmental methods of combating the pandemic. It should also be noted that users of social networks cannot identify fake news or trust unverified information. Therefore, the experiment conducted on the topic of vaccination against the coronavirus disease makes it possible to understand the public’s attitude and the Government’s activities by assessing comments’ SA and semantic content. As a result, it will make it possible to maintain an exploratory policy for the public correctly, determine the presentation style of information material, accelerate the introduction of such large-scale state tasks, and ensure the preservation of public health. Furthermore, the OMSystem is used as a serious analytics tool to estimate the user perception of social life, which will allow quick explanations for the public, identify alarming factors of the public, and evaluate social mood.

## Conclusion

A comparative analysis of foreign analytics platforms and the developed Kazakhstani OMSystem made it possible to conclude that foreign analytics platforms are mostly aimed at business and brand promotion. At the same time, they cover only the information space of foreign countries and are little focused on existing social problems. The existing iMAS, Alem Media Monitoring, and our OMSystem analytics platforms of Kazakhstan pay more attention to the analysis of public opinion on a wide range of political and socio-economic problems. They aim to cover the most relevant topics over large and small-time ranges and use ML algorithms to quickly and efficiently determine the sentiment of texts and user comments. The OMSystem monitors the current political and socio-economic situation in the country, allows searching for the keywords on any desired topics, defines topics’ sentiment with the dictionary and ML algorithms approaches, and determines the social well-being based on such indicators as the level of topic discussion activity in society, the level of interest in the topic in society, and the level of social mood. In this paper, the functionalities of the main modules of the OMSystem, such as the ‘Connector module,’ the ‘Linguistic constructor module,’ the ‘Data analysis and processing module,’ and the ‘Results module’ were thoroughly investigated. The formation of the Russian and Kazakh datasets was described. Then the text preprocessing, stemming, vectorization, and class resampling techniques were shown. In order to label the texts on their emotional aspects, NB, LR, SVM, k-NN, DT, RF, and XGBoost ML algorithms were used to train the models. The performance of the models was evaluated by the accuracy, precision, recall, and F1-score metrics. Among all the conducted experiments, DT and RF showed the best results reaching an accuracy of 0.95–0.99 with the Random oversampling techniques. These models are added to the OMSystem. The second part of the experiments analyzed the social mood on the topic of vaccination against the coronavirus disease. The use of the social analytics metrics: the level of interest in the topic in society, the level of topic discussion activity in society, and the level of social mood made it possible to understand the public’s attitude and the Government’s activities with the summary tables, graphics, and plots. The OMSystem will also be used for the evaluation of the user perception of the important and relevant topics in future works.

## Data Availability

The datasets for training ML models are available at the following link (https://drive.google.com/file/d/1eAmVsYifgpkATw-Xl_Agjqxesr3VQF0U/view?usp=sharing). The database of the experimental results on the social mood on the topics related to Covid-19 of the OMSystem, corresponding pivot tables in the Russian language, and their translation to the English language and graphics are available from the corresponding author on the request.

## Abbreviations

SA: Sentiment analysis
ML: Machine learning
SVM: Support vector machine
NB: Naïve Bayes
k-NN: K-nearest neighbors
LR: Logistic regression
DT: Decision tree
FR: Random forest
CNN: Convolutional neural networks
RNN: Recurrent neural networks
LSTM: Long short-term memory
REST API: Representational state transfer application programming interface
APIs: Application programming interfaces
SMMM: Social media marketing management
